# Dynamics of *Wolbachia pipientis* Gene Expression Across the *Drosophila melanogaster* Life Cycle

**DOI:** 10.1534/g3.115.021931

**Published:** 2015-10-23

**Authors:** Florence Gutzwiller, Catarina R. Carmo, Danny E. Miller, Danny W. Rice, Irene L. G. Newton, R. Scott Hawley, Luis Teixeira, Casey M. Bergman

**Affiliations:** * Faculty of Life Sciences, University of Manchester, M13 9PT Manchester, United Kingdom; † Instituto Gulbenkian de Ciência, P-2780-156 Oeiras, Portugal; ‡ Stowers Institute for Medical Research, Kansas City, Missouri 64110; § Department of Molecular and Integrative Physiology, University of Kansas Medical Center, Kansas City, Kansas 66160; ** Department of Biology, Indiana University, Bloomington, Indiana 47405

**Keywords:** *Wolbachia*, *Drosophila*, symbiosis, development, cytoplasmic incompatibility

## Abstract

Symbiotic interactions between microbes and their multicellular hosts have manifold biological consequences. To better understand how bacteria maintain symbiotic associations with animal hosts, we analyzed genome-wide gene expression for the endosymbiotic α-proteobacteria *Wolbachia pipientis* across the entire life cycle of *Drosophila melanogaster*. We found that the majority of *Wolbachia* genes are expressed stably across the *D. melanogaster* life cycle, but that 7.8% of *Wolbachia* genes exhibit robust stage- or sex-specific expression differences when studied in the whole-organism context. Differentially-expressed *Wolbachia* genes are typically up-regulated after *Drosophila* embryogenesis and include many bacterial membrane, secretion system, and ankyrin repeat-containing proteins. Sex-biased genes are often organized as small operons of uncharacterized genes and are mainly up-regulated in adult *Drosophila* males in an age-dependent manner. We also systematically investigated expression levels of previously-reported candidate genes thought to be involved in host-microbe interaction, including those in the WO-A and WO-B prophages and in the Octomom region, which has been implicated in regulating bacterial titer and pathogenicity. Our work provides comprehensive insight into the developmental dynamics of gene expression for a widespread endosymbiont in its natural host context, and shows that public gene expression data harbor rich resources to probe the functional basis of the *Wolbachia-Drosophila* symbiosis and annotate the transcriptional outputs of the *Wolbachia* genome.

Intracellular bacterial symbioses provide powerful systems to investigate the diverse consequences of coevolution between microbes and their hosts. Some bacterial endosymbiotic interactions are beneficial to both organisms, resulting in coadapations that generate “obligate” dependency. Obligate symbioses are often characterized by ancient phylogenetic associations, restriction of microbes to specialized host cells, provision of essential nutrients from microbe to host, and extreme microbial genome reduction (reviewed in Dale and Moran 2006; Moran *et al.* 2008). Other microbial endosymbiotic interactions are obligate for the microbe, but are nonessential (“facultative”) from the standpoint of the host. Facultative endosymbionts are of particular interest since some may represent a transitional state between free-living bacteria and obligate mutualists, thus offering insights into both the early evolutionary stages of mutualism and the propagation of invasive pathogens (Dale and Moran 2006; Moran and Degnan 2006).

Efforts to identify microbial genes that maintain infections of facultative endosymbionts are hampered by the inability to culture and manipulate these species in a free-living state. Likewise, the lack of extreme genome reduction in facultative endosymbionts does not allow the mere existence of a gene to provide *prima facie* evidence of its importance in a particular host context, as it does in mutualist species with highly-reduced genomes (Moran and Degnan 2006). Therefore, candidate genes in facultative endosymbionts that might mediate interaction with their hosts have been primarily identified using comparative genomic approaches. For example, initial sequencing of the *Wolbachia pipientis* genome from the arthropod *Drosophila melanogaster* revealed an unusually large number of ankyrin repeat domain (ANK) encoding genes relative to other bacteria (Wu *et al.* 2004). Large numbers of ANK-containing genes are also observed in the genomes of other *Wolbachia* strains that form facultative associations with arthropod hosts (Iturbe-Ormaetxe *et al.* 2005; Duron *et al.* 2007; Siozios *et al.* 2013), while few ANK-containing genes are found in the obligate *Wolbachia* endosymbionts of nematodes (Foster *et al.* 2005; Darby *et al.* 2012). Comparative genomic analysis of more closely-related strains of *Wolbachia* has also been used to identify candidate genes involved in host-symbiont interaction (Iturbe-Ormaetxe *et al.* 2005; Sinkins *et al.* 2005; Duron *et al.* 2007; Chrostek *et al.* 2013; Woolfit *et al.* 2013). For example, a cluster of eight genes (called the Octomom region), identified as being specifically duplicated in the pathogenic “Popcorn” (*w*MelPop) strain of *Wolbachia* from *D. melanogaster* (Chrostek *et al.* 2013; Woolfit *et al.* 2013), was recently shown to cause the high bacterial titers and virulence associated with this strain (Chrostek and Teixeira 2015).

Genome-wide gene expression profiling offers another promising approach to identify candidate genes involved in host-symbiont interactions. Both transcriptomics and proteomics have been used successfully to study how bacterial gene regulation changes in native host tissues for obligate endosymbionts (Wilcox *et al.* 2003; Moran *et al.* 2005; Reymond *et al.* 2006; Bennuru *et al.* 2011; Darby *et al.* 2012; Rao *et al.* 2012; Luck *et al.* 2014). However, genome-wide expression profiling has not yet been used extensively to study gene expression dynamics for facultative endosymbionts in their native host context (Slatko *et al.* 2014). Recently, Darby *et al.* (2014) conducted transcriptomic and proteomic analysis of a *Wolbachia* strain from *D. melanogaster* (*w*MelPop-CLA) and Baldridge *et al.* (2014) profiled the proteome of *Wolbachia* wStr from the planthopper *Laodelphax striatellus*. Both of these studies used stably transinfected nonnative host cell lines from the mosquito *Aedes albopictus*. Likewise, two recent studies have also used *w*MelPop-CLA transfected in nonnative *Aedes* cell lines to identify small noncoding RNAs (ncRNAs) by high-throughput sequencing (Mayoral *et al.* 2014; Woolfit *et al.* 2015). Woolfit *et al.* (2015) also generated transcriptomic data from two *Wolbachia* strains (*w*MelPop and *w*MelCS) in native host tissues (heads of *D. melanogaster*), but did not attempt to identify differentially expressed genes that may be involved in host-microbe interactions.

Here, we report global gene expression dynamics for a facultative endosymbiont across the life cycle of a native arthropod host. The rationale for this analysis is to identify bacterial genes involved in maintaining facultative endosymbiotic associations on the basis of their differential expression across host life-cycle stages. Our work takes advantage of a previously uncharacterized *Wolbachia* infection in the ISO1 reference strain that was used for the *D. melanogaster* genome project (Brizuela *et al.* 1994; Adams *et al.* 2000). We show that the *D. melanogaster* ISO1 strain was originally infected with *Wolbachia* prior to being donated to the *Drosophila* stock center, whereafter it was used by the modENCODE project to generate deep total RNA-seq data from 30 time points across the *D. melanogaster* life cycle including embryos, larvae, pupae, adult males, and adult females (Graveley *et al.* 2011; Brown *et al.* 2014; Duff *et al.* 2015). Using this rich transcriptomic resource, we show that the majority of *Wolbachia* genes are expressed across the life cycle, but that most *Wolbachia* genes show stable expression across different host stages and sexes when studied at the whole-fly level. We identify a set of 80 genes that show reproducible changes in expression levels in at least one life-cycle stage, the majority of which are up-regulated after embryonic development with peaks of expression in early larval, late pupal or adult stages. We also identify 41 genes that show expression differences between adult males and females, with the majority of these sex-biased genes being up-regulated in males and showing age-dependent effects. Genes with stage- or sex-specific expression differences include chaperones, ANK-containing genes, and genes with predicted membrane or secretion system function, but most have no known function. Our results provide general insight into the dynamics of gene expression in a facultative endosymbiont across different life cycle stages and sexes of an arthropod host, and provide a rich set of resources to further explore the functional basis of the *Wolbachia-Drosophila* symbiosis.

## Materials and Methods

### *D. melanogaster* strains and husbandry

Substrains of the *D. melanogaster* ISO1 strain originally described in Brizuela *et al.* (1994) were obtained from several sources: (i) the Bloomington *Drosophila* Stock Center (BDSC ISO1, stock #2057); (ii) Jim Kennison (National Institute of Child Health and Human Development); (iii) Todd Laverty (Howard Hughes Medical Institute Janelia Farms); and (iv) Sue Celniker (Lawrence Berkeley National Laboratory). The construction of the isogenic line carrying *Wolbachia* variant *w*Mel in a DrosDel w1118 background (Ryder *et al.* 2004) is described in Chrostek *et al.* (2013). *D. melanogaster* lines were maintained on a standard cornmeal diet at a constant temperature of 25°.

We generated versions of all ISO1 substrains cured of any potential *Wolbachia* infection by treating with tetracycline for two generations. Adults were allowed to lay eggs for 5 d on Formula 4-24 food (Carolina, cat #173210) mixed with equal part water containing 0.25 mg/ml tetracycline. Offspring from the first generation were collected at 10 d and transferred to new food containing 0.25 mg/ml tetracycline and allowed to lay eggs for 5 d. Offspring from the second generation were collected at 10 d and transferred onto standard cornmeal-agar food to establish *Wolbachia*-free stocks.

### *Wolbachia* infection status

DNA for polymerase chain reaction (PCR) screening of *Wolbachia* infection status was prepared from single flies by placing individual males in a standard fly squish buffer (50 μl of 1M Tris pH 8.0, 0.5M EDTA, 5M NaCl) plus 1 μl of 10 mg/ml Proteinase K. Flies were then placed in a thermocycler at 37° for 30 min, 95° for 2 min followed by a 4° hold. PCR was performed using 4 μl of fly squish product in a total volume of 50 μl. The presence of *Wolbachia* was confirmed by PCR using two sets of primers: (i) Wolbachia_F2 (5′-TGGCTCACATAGATGCTGGT- 3′) and Wolbachia_R2 (5′-GTCCCATTTCTCACGCATTT-3′); and (ii) Wolbachia_F3 (5′-ATCCTGCAAATTGGCGTACT-3′) and Wolbachia_R3 (5′-ATAACGCACACCTGGCAAAT-3′). To ensure DNA preparation was sufficient for PCR amplification, control primers were used from the *D. melanogaster* genome: rDNA-F (5′-AAACTAGGATTAGATACCCTATTAT-3′) and rDNA-R (5′-AAGAGCGACGGGCGATGTGT-3′). PCR was performed with Kappa HiFi polymerase (KAPA Biosystems, KK2502) using the following reaction conditions: 30 cycles of 95° for 20 sec, 60° for 15 sec, and 72° for 90 sec.

### Genome sequencing and data analysis

Genomic DNA for the BDSC ISO1 strain was prepared from 10 starved, adult males using the Qiagen DNeasy Blood and Tissue Kit (Qiagen, 69504). A total of 1 μg of DNA was fragmented using a Covaris S220 sonicator (Covaris Inc.) to 250 bp fragments by adjusting the treatment time to 85 sec. Following the manufacturer’s directions, short fragment libraries were made using KAPA Library Preparation Kits (KAPA Biosystems, KK8201) and Bioo Scientific NEXTflex DNA Barcodes (Bioo Scientific, 514104). The resulting libraries were purified using the Agencourt AMPure XP system (Beckman Coulter, A63880), then quantified using a Bioanalyzer (Agilent Technologies) and a Qubit Fluorometer (Life Technologies). Libraries were pooled with other strains, requantified, and run for 100 cycles in paired-end high output mode over multiple lanes on an Illumina HiSeq 2000 instrument using HiSeq Control Software v1.5.15.1 and Real-Time Analysis v1.13.48.0. CASAVA v1.8.2 was run to demultiplex reads and generate fastq files.

Fastq sequences were mapped against a “holo-genome” consisting of the Release 5 version of the *D. melanogaster* genome (Ensembl Genomes Release 24, Drosophila_melanogaster.BDGP5.24.dna.toplevel.fa) and the *Wolbachia w*Mel reference genome (Ensembl Genomes Release 24, Wolbachia_endosymbiont_of_drosophila_melanogaster.GCA_000008025.1.24) (Cunningham *et al.* 2015; Kersey *et al.* 2014). Holo-genome reference mapping was performed using bwa mem v0.7.5a (Li 2013) with default parameters in paired-end mode. Mapped reads for all runs from the same sample were merged, sorted and converted to BAM format using samtools v0.1.19 (Li *et al.* 2009). BAM files were then used to create BCF and fastq consensus sequence files using samtools mpileup v0.1.19 (options -d 100000). Fastq consensus sequence files were converted to fasta using seqtk v1.0-r76-dirty (https://github.com/lh3/seqtk) and concatenated with consensus sequences of *Wolbachia*-type strains from Chrostek *et al.* (2013). Maximum-likelihood phylogenetic analysis on resulting multiple alignments was performed using raxmlHPC-PTHREADS v8.1.16 (options -T 6 -f a -x 12345 -p 12345 -N 100 -m GTRGAMMA) (Stamatakis 2014).

### RNA-seq data analysis

We analyzed total RNA-seq libraries from the modENCODE developmental time course, which samples 30 time points from the BDSC ISO1 substrain across the *D. melanogaster* life cycle including embryos, larvae, pupae, adult males, and adult females. Information about the RNA sample collection (Graveley *et al.* 2011; Brown *et al.* 2014) and RNA-seq library construction and sequencing (Duff *et al.* 2015) for these datasets has been described previously. Total RNA-seq libraries analyzed are 100 bp read length, rRNA-depleted, paired-end, and stranded, with two biological replicates available for 24 of the 30 time points. All nonadult samples are from mixed sex organisms in unknown ratios; adult female samples are from mated and virgin flies in unknown ratios.

Total RNA-seq fastq sequences from SRP001696 were downloaded and mapped against the holo-genome described above in paired-end mode using bwa mem v0.7.5a with default parameters (accession numbers for samples used in this study are given in Supporting Information, Table S1). Resulting mapped reads were sorted and converted to BAM format using samtools v0.1.19. Counts for both forward and reverse reads together were used to summarize numbers of reads mapping to the *Wolbachia* and *D. melanogaster* genomes. Forward reads from each read-pair (which correspond to the antisense orientation in the Illumina TruSeq Stranded Total RNA kit used) were converted to the opposite strand and combined with reverse reads to generate wiggle plots of strand-specific RNA-seq coverage.

Sorted BAM files were used to count reads overlapping protein-coding genes on the sense orientation by one or more bp using BEDtools v2.22.0 (Quinlan and Hall 2010) with the Ensembl Genomes Release 24 version of the *Wolbachia* genome annotation (Wolbachia_endosymbiont_of_drosophila_melanogaster.GCA_000008025.1.24.gtf). We did not attempt to estimate expression levels for annotated ncRNA genes because bacterial rRNA transcripts were not targeted for depletion by modENCODE and thus *Wolbachia* rRNA expression levels are high and variable among samples, which affects normalization of all protein-coding genes. Since current gene models in *Wolbachia* correspond only to coding regions and not full-length transcripts, we chose a read counting strategy that allowed RNA-seq reads to extend beyond currently-annotated gene model limits. Only counts for the reverse read from each read-pair (which corresponds to the sense orientation in the Illumina TruSeq Stranded Total RNA kit) were used for expression level estimates, differential expression analysis, and clustering.

We performed differential expression analysis using edgeR v3.6.8 (Robinson *et al.* 2010) with p-values adjusted using the method of Benjamini and Hochberg (1995) to correct for multiple testing. Read counts were normalized using the trimmed mean of M-values method (Robinson and Oshlack 2010) and models were fitted using tagwise dispersion (Robinson and Smyth 2007). To identify *Wolbachia* genes that change in any stage across the life cycle, we performed a single analysis using an ANOVA-like generalized linear model approach (McCarthy *et al.* 2012) using all stages of the ISO1 developmental time course that had replicates (24 time points) with an adjusted p-value cutoff of 0.05. To identify *Wolbachia* genes that change between pairs of samples we used an exact test approach (Robinson and Smyth 2008) with adjusted p-value 0.01 and twofold change cutoffs.

Probabilistic clustering on all samples from the ISO1 time course (both with and without biological replicates) was performed with MBcluster.seq v.1.0 (Si *et al.* 2014) using the Poisson model with two clusters and the expectation maximization method. Because MBcluster.seq is a probabilistic method, we performed 1000 runs of the clustering analysis. We matched cluster identifiers from different runs using the fact that the majority of genes are stably expressed across the life cycle, and defined the cluster with the majority of genes as “cluster 1” and the remaining genes as “cluster 2”. Genes assigned to cluster 2 were further classified into subclusters 2a and 2b on the basis of the number of runs in which a gene was assigned to cluster 2.

Within-sample normalized read counts in units of transcripts per million (TPM) (Li and Dewey 2011) were also used to generate between-sample correlation and gene-by-sample heatmaps. Effective gene length in TPM normalization was set to be gene_length+read_length−1 to account for reads that extend beyond annotated gene models. Normalization by library size in TPM and differential expression analyses removes the ability to detect global up- or down-regulation of all *Wolbachia* genes that might occur from differences in *Wolbachia* titer among samples, but does not affect the ability to detect difference among samples due to differential expression of individual genes. Differential expression, clustering, and visualization were performed using R software v3.1.1 (R Development Core Team 2012).

### Reverse transcription real-time quantitative PCR

RNA for reverse transcription real-time quantitative PCR (RT-qPCR) was obtained from embryos, adult males, and adult virgin females for the BDSC ISO1 and DrosDel w1118 strains. Two independent collections, each with five biological replicates per stage, were performed for each *D. melanogaster* line. For embryo collection, flies laid eggs for 2 hr on agar plates supplemented with 1:1 yeast/water paste. After 16 hr at 25° the embryos were collected, treated with 2% sodium hypochlorite, and washed with sterile water before RNA extraction. A total of 500 embryos were used per sample. For adult collection, males and females were separated immediately after eclosion and maintained on a standard diet for 24 hr before RNA extraction. Ten adult flies were used per sample. Samples were homogenized with a plastic pestle in 1 ml of Trizol Reagent (Ambion, 15596-018). RNA was extracted according to the manufacturer’s protocol and resuspended in 50 μl of diethylpyrocarbonate-treated water (Ambion, AM9915G). RNA concentrations were determined using a NanoDrop ND-1000 Spectrophotometer. cDNA was prepared from 4 μg of total RNA using random primers (Promega, C1181) and Moloney Murine Leukemia Virus reverse transcriptase (Promega, M1705). Primers were allowed to bind to the template RNA at 70° for 5 min and the reaction proceeded at 25° for 10 min, 37° for 60 min, and 85° for 10 min.

RT-qPCR reactions were carried out in a CFX384 Real-Time PCR Detection System (Bio-Rad). Reactions were carried out in 384-well plates (Bio-Rad, HSP3805) using iTaq Universal SYBR Green Supermix (Bio-Rad, 172-5125), 0.15 μM of each primer, and 5 μl of cDNA diluted 1:50 in water. Each complete, independent collection of each *D. melanogaster* line was analyzed in one plate. Each plate contained two technical replicates of every sample for each set of primers. Sequences of the primers used for RT-qPCR can be found in Table S4. Amplification conditions were set up as follows: 50° for 2 min, 95° for 10 min, followed by 40 cycles of 95° for 30 sec, 57° for 1 min, and 72° for 30 sec. Melting curves were analyzed to confirm the specificity of amplified products and Ct values were obtained using Bio-Rad CFX Manager default threshold settings. Relative transcript expression levels were calculated by the method of Pfaffl (2001). Gene expression was normalized using as reference genes the three stably-expressed *Wolbachia* genes WD1043, WD1063, and WD1071, which were selected because they exhibit low fold-change and low coefficient of variation across the ISO1 life cycle time course (de Jonge *et al.* 2007). Expression values were calculated relative to embryonic expression levels.

Relative gene expression values were analyzed using R software v3.1.1 (R Development Core Team 2012) by fitting a linear mixed-effect model to the data of each gene using the lmer package (v2.0-20), comparing the effect of stage (embryo, adult male, and adult female) with a Tukey’s all-pair comparison using the glht package (v1.3-9). The data of the two genotypes were analyzed separately and together. For the linear mixed-model, the stage and genotype (in the joint analysis) were considered fixed effects, while independent collection was considered a random effect. No correction was applied to p-values, and thus α-levels for significance were set at 0.001 to account for multiple testing.

### Functional and comparative annotation of *Wolbachia* genes

We generated functional annotations for *Wolbachia* genes using three sources: (i) by querying *w*Mel open reading frames against the Genbank nucleotide (nt) database (April 2012, 15,938,872 sequences; 40,783,330,152 letters) using TBLASTN v2.2.25+ (Altschul *et al.* 1997) with default options; (ii) by querying *w*Mel open reading frames against the Pfam-A.hmm database (v26.0) (Finn *et al.* 2014) using hmmscan v3.0 with default options (http://hmmer.org); and (iii) by using the original functional annotations generated by TIGR.

We identified homologs of *w*Mel genes by conducting an all-*vs.*-all search of genes from the following complete *Wolbachia* genomes using BLASTP 2.2.27+ with default options: *w*Ri (supergroup A strain from *D. simulans*, NC_012416), *w*Pip-Pel (supergroup B strain from *Culex quinquefasciatus*, NC_010981), and *w*Bm (supergroup D strain from *Brugia malayi*, NC_006833). The best hit to a gene in genome A was defined as the gene in genome B that had the highest bit score. Homology groups were defined such that a member had to have a reciprocal best hit to at least one other member of the group (single linkage), which permits paralogs to be included in a group.

### Data availability

DNA-seq fastq reads for BDSC ISO1 were submitted to European Nucleotide Archive as experiment ERX645969. Table S1 contains a summary of the number of mapped RNA-seq reads for *D. melanogaster* and *Wolbachia*, number of expressed genes (defined as genes with nonzero TPM or genes with ≥2 mapped reads per gene), mean TPM for the sample (same for all samples, inverse of gene number times one million), and standard deviation of TPM for the sample for each total RNA sample in SRP001696. Table S2 contains gene IDs, coordinates, number of reads (from read 2 of paired-end data) mapping to the sense strand in each sample (_r1 = replicate 1, _r2 = replicate 2), estimated TPM for each sample, number of runs found in cluster 2, cluster assignment, adjusted p-value in life cycle GLM, log_2_ fold-change in life cycle GLM *vs.* embryo 0-2 hr, maximum fold change between any two stages in life cycle GLM, adjusted p-values and log_2_ fold change for pairwise exact tests between male and female samples at 1, 5, and 30 d, gene name, annotated gene product, effective number of codons, GC content, and number of homologs in *w*Mel, wRi, *w*Pip-Pel, and *w*Bm genomes. Table S3 contains RT-qPCR GLM analysis results for ISO1 and DrosDel w1118. The p-values reported in Table S3 are not adjusted for multiple testing, and thus α-levels for significance were set at 0.001. Table S4 contains sequences of PCR primers used for RT-qPCR experiments.

## Results and Discussion

### The *D. melanogaster* ISO1 reference strain is infected with *Wolbachia*

As a control for another project, we obtained the ISO1 reference strain (Brizuela *et al.* 1994) used for the *D. melanogaster* genome project from the Bloomington *Drosophila* Stock Center (BDSC) and sequenced its genome. We discovered that the BDSC ISO1 sample contained a large number of *Wolbachia* sequences (4.5 million reads, 2.5% of total) when mapped against a “holo-genome” comprised of the *D. melanogaster* plus *W. pipientis w*Mel reference genomes (Adams *et al.* 2000; Wu *et al.* 2004). The observation of *Wolbachia* sequences in the ISO1 stock was unexpected, since at no point since its original sequencing by the Berkeley *Drosophila* Genome Project (BDGP) and Celera Genomics in 2000 had *Wolbachia* sequences been reported in this strain (Adams *et al.* 2000; Celniker *et al.* 2002; Hoskins *et al.* 2015). In fact, direct searches of assembled or unassembled ISO1 genomic sequences from the BDGP failed to detect any evidence of *Wolbachia* (Wu *et al.* 2004; Salzberg *et al.* 2005). By investigating the provenance and conducting PCR-based assays of *Wolbachia* infection status of a panel of ISO1 substrains (see details in File S1), we confirmed that the BDSC ISO1 substrain is indeed infected with *Wolbachia* and established that loss of the *Wolbachia* infection occurred on the lineage leading to the BDGP ISO1 substrain.

**Figure 1:**
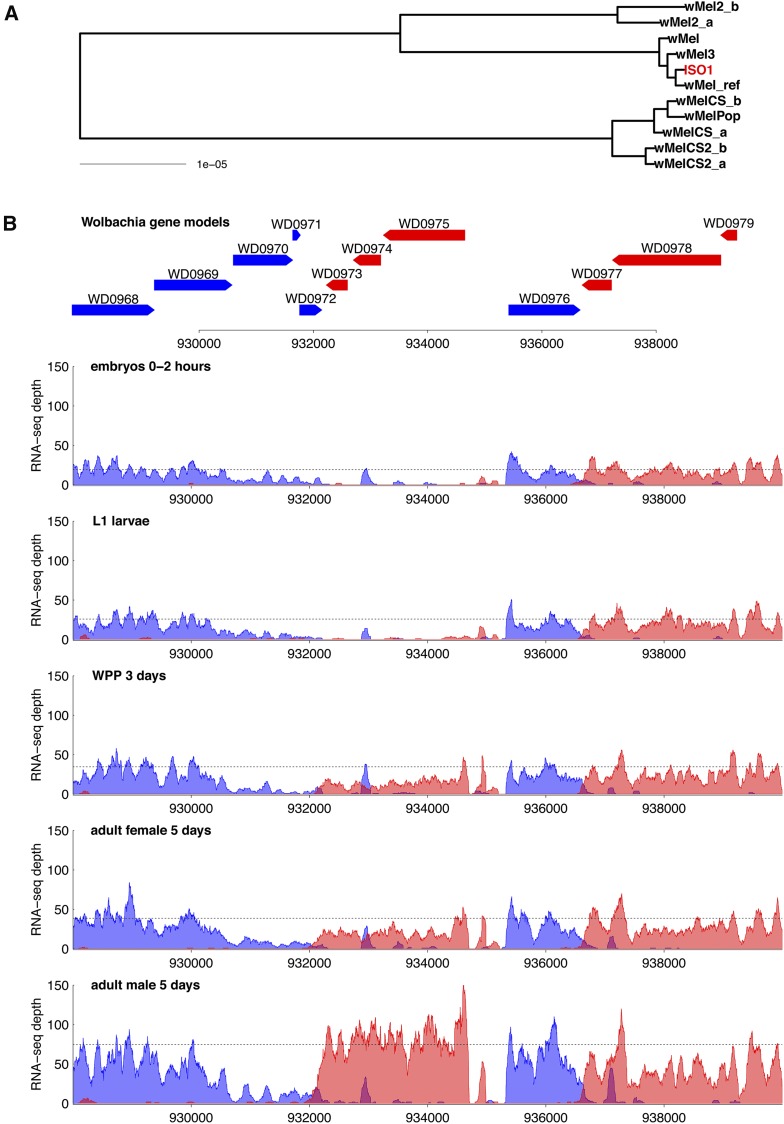
Phylogeny and expression landscape of *Wolbachia* in *D. melanogaster*. (A) Phylogenetic tree of *Wolbachia* strains based on whole genome sequences from this study (ISO1, red), Wu *et al.* (2004) (*w*Mel_ref), and Chrostek *et al.* (2013) (all others). The scale bar for branch lengths is units of substitutions per site. The *Wolbachia* variant in ISO1 is very closely related to the *w*Mel reference genome and to the *w*Mel variant from Chrostek *et al.* (2013). (B) Gene models and RNA-seq coverage plots for a 12-gene window of the *Wolbachia* genome showing gene expression levels in representative stages of the *D. melanogaster* modENCODE RNA-seq life cycle time course. Gene models (pointed rectangles) and RNA-seq coverage (strand-specific wiggle plots of number of reads mapped to each base pair) are shown on the forward and reverse strands in blue and red, respectively. RNA-seq plots are shown on the same absolute *y*-axis scale. To provide an internal normalization factor for comparison across samples, mean coverage of the stably-expressed Wsp/WD1063 gene (not shown in this interval) divided by 20 is depicted by the dashed line in each panel. This example shows a set of three consecutive genes (WD0973, WD0974 and WD0975) that are cotranscribed as a single operon and specifically up-regulated in males in comparison to neighboring genes, as well as an unannotated noncoding RNA transcript that is expressed antisense to the 3′-end of WD0974.

We next addressed which of the major variants of *Wolbachia* that are known to exist in *D. melanogaster* infects the ISO1 reference strain (Richardson *et al.* 2012; Chrostek *et al.* 2013; Woolfit *et al.* 2013). To do this, we assembled a consensus sequence from BDSC ISO1 reads that mapped to the *w*Mel reference, then generated a whole-genome phylogeny jointly with the *w*Mel reference genome (Wu *et al.* 2004) and genomes from known *Wolbachia* genotypes (Chrostek *et al.* 2013). This analysis showed that the *Wolbachia* infection in the BDSC ISO1 substrain is from a *w*Mel-like genotype that is very closely related to both the *w*Mel reference genome sequence (Wu *et al.* 2004) and the *w*Mel-type strain recently reported by Chrostek *et al.* (2013) (Figure 1A). The very high sequence similarity between the *w*Mel genotype in ISO1 and the *w*Mel reference genome allows functional genomic data collected in ISO1 to be easily and accurately mapped to the reference genome sequence, and implies that *w*Mel reference genome annotations closely reflect the content of the ISO1 *Wolbachia* genome.

### The modENCODE developmental time course reveals a small subset of *Wolbachia* genes with dynamic expression across the *D. melanogaster* life cycle

Establishing that the BDSC ISO1 substrain is infected with *Wolbachia* is important since this strain is widely used in *Drosophila* genomics, including being one of the strains used by modENCODE to profile the transcriptome of *D. melanogaster* (Graveley *et al.* 2011; Brown *et al.* 2014; Duff *et al.* 2015). In particular, modENCODE-generated total RNA-seq libraries from BDSC ISO1 that span 30 time points across the *D. melanogaster* life cycle including multiple stages from embryos, larvae, pupae, and both adult sexes, with two biological replicates being available for 24 of the 30 time points (Graveley *et al.* 2011; Brown *et al.* 2014; Duff *et al.* 2015). We tested whether the *Wolbachia* infection in ISO1 could be detected in modENCODE total RNA-seq libraries by mapping reads to the combined *D. melanogaster* plus *W. pipientis* holo-genome reference. We found that the modENCODE total RNA-seq libraries do contain large numbers of *Wolbachia* sequences, with a median of 1.7 million reads per sample (range: 0.1–7 million) mapping to the *Wolbachia* genome, corresponding to a median of 1.6% (range: 0.3%–8.5%) of the total number of RNA-seq reads mapped in each sample (Table S1). As shown in Figure 1B, coverage and strand-specificity of the modENCODE RNA-seq dataset is high enough to show clear correspondence with the boundaries of most annotated *Wolbachia* gene models, given their presumed operonic structure and lack of annotated untranslated regions. Analysis of modENCODE RNA-seq libraries showed that the majority of *Wolbachia* genes were expressed in each sample, and that expression levels of genes were highly correlated among biological replicates (Figure S1 and File S1). These results further confirm that the BDSC ISO1 substrain is indeed infected with *Wolbachia*, and allow the modENCODE RNA-seq developmental time course to be analyzed in the context of the *Wolbachia-Drosophila* symbiosis.

We exploited our observation that modENCODE time course contains an essentially-complete *Wolbachia* transcriptome to study how *Wolbachia* expression varies across the *D. melanogaster* life cycle. Globally, we saw high correlations in expression levels across all stages (Figure S1 and File S1), with two weakly-differentiated, partially-overlapping clusters spanning embryonic to white prepupal (WPP) stages, and late larval to adult stages, respectively. In addition to the larger embryonic/pupal and pupal/adult clusters, stage-specific clusters could be observed for embryonic 10–12 hr, larval L1, larval L2, and larval L3 samples. Genome-wide expression changes at these particular stages suggest a potential link to pulses of ecdysone, a steroid hormone that regulates many aspects of arthropod development, which has been implicated in mediating the phenotypic effects of *Wolbachia* on its hosts (Negri 2012).

**Figure 2:**
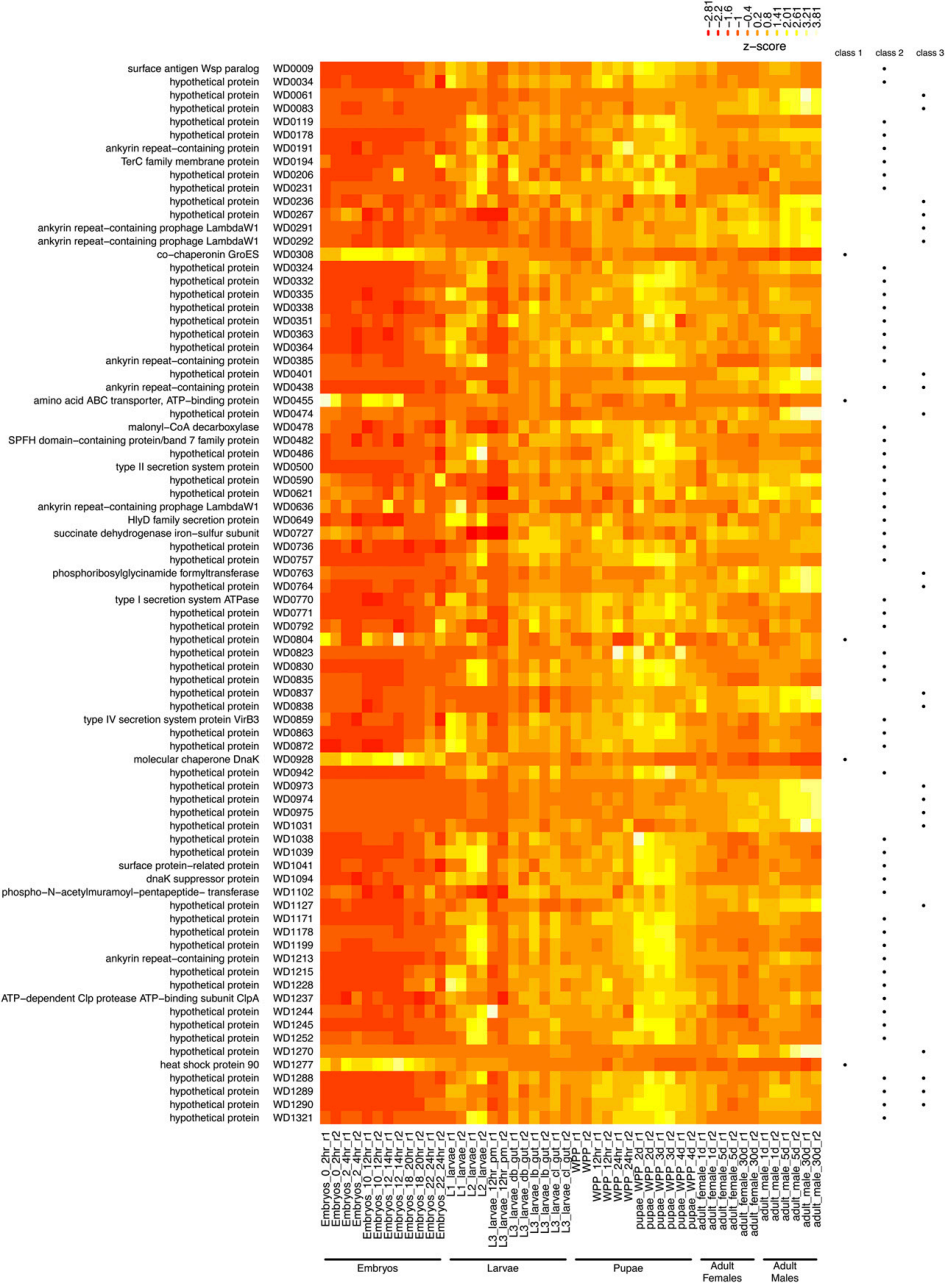
A small subset of *Wolbachia* genes show differential expression across the *D. melanogaster* life cycle. Row-normalized expression levels are visualized as a heatmap where each row represents a gene (ordered top-to-bottom by its position in the genome) and each cell represents the relative expression level for a particular sample in terms of Z-scores [observed transcripts per million (TPM) minus row mean TPM, divided by the standard deviation of TPMs for that row]. Values higher than row means are represented by yellow, and values lower than row means are represented by red. Gene names and identifiers are shown on the left. Membership in dynamically-expressed gene classes is shown by dots on the right. Class 1 includes genes that show down-regulation after embryogenesis. Class 2 includes genes that show up-regulation after embryogenesis, with peaks of expression in larval and pupal stages. Class 3 includes genes that show up-regulation after embryogenesis, with peaks of expression in adults. Classification of gene sets is not mutually exclusive. Stages that lack biological replicates in the modENCODE total RNA-seq time course were not used in this analysis and are not shown here.

To identify specific genes whose expression levels vary reproducibly across life cycle stages, we performed differential expression analysis across all 24 stages that had biological replicates using an ANOVA-like GLM approach (McCarthy *et al.* 2012). We chose to perform an omnibus test of changes in expression across life cycle stages simultaneously because of the large number of life cycle stages and their complex developmental dependencies. This analysis revealed a small subset of *Wolbachia* genes (80/1195, 6.7%) that were differentially expressed in one or more life cycle stage at an adjusted p-value of less than 0.05 (Figure 2 and Table S2). All 80 genes had a greater than twofold change between at least one pair of stages. The vast majority of *Wolbachia* genes identified as differentially expressed across the *D. melanogaster* life cycle in the modENCODE time course showed a common pattern of being expressed at lower relative levels in embryos (75/80, 93.8%), with higher expression in either larval, pupal, and/or adult stages, and a transient decrease in expression at larval L3 (12 hr). However, five genes show the opposite pattern of having higher relative expression in embryos with down-regulation later in the life cycle (GroES/WD0308, ABC transporter/WD0455, WD0804, DnaK/WD0928, and Hsp90/WD1277). The dynamics of up-regulated and down-regulated genes show nearly complementary transitions at the end of embryogenesis, suggesting a response to common signals or possible cross-talk between these gene sets. We note that both up- and down-regulated genes exhibit a wide range of absolute expression levels, and many show quantitative shifts rather than dramatic qualitative changes in expression level.

To support conclusions about the global pattern of *Wolbachia* gene expression dynamics across the *D. melanogaster* life cycle based on differential expression analysis, we performed RT-qPCR for a sample of 10 genes in ISO1 and a second *D. melanogaster* strain (DrosDel w1118) carrying a *w*Mel infection (Figure S2 and File S1). RT-qPCR results validated the main *Wolbachia* gene expression dynamics inferred from whole-organism RNA-seq in *D. melanogaster*, and indicated that gene expression information from the ISO1 RNA-seq time course can likely be extrapolated to other strains carrying *w*Mel-like *Wolbachia* infections. We also performed probabilistic clustering on the entire modENCODE RNA-seq time course (including stages without replicates) (Si *et al.* 2014) (Figure S3 and File S1). This analysis identified two main clusters that could be matched across independent clustering runs (Figure S3A and Table S2). The first cluster contained the majority of *Wolbachia* genes (1033, 86.4%) and showed a pattern of relatively stable expression levels across the life cycle (Figure S3B). The second cluster contained the remaining 162 genes (13.6%), which generally showed up-regulation after embryogenesis and included the vast majority of genes identified as differentially expressed in the life cycle GLM (74/80, 92.5%). Overall, clustering analysis supported the main conclusions of the differential expression analysis that only a small proportion of *Wolbachia* genes show robust differences in expression across the modENCODE life cycle time course at the level of the whole organism, and that the majority of dynamically-expressed *Wolbachia* genes show up-regulation after embryogenesis. However, the greater number of genes identified as being dynamically expressed by clustering relative to the life cycle GLM suggests that our differential expression analysis may have detected only a conservative subset of *Wolbachia* with the strongest expression differences across *D. melanogaster* development.

### Dynamically-expressed *Wolbachia* genes are predicted to be involved in stress response and host-microbe interactions

The 80 *Wolbachia* genes that exhibited dynamic expression across the modENCODE *D. melanogaster* life cycle time course fall into three broad classes (Figure 2). The first is a small class of five genes that show high relative expression in embryos with down-regulation later in the life cycle. Three of these genes are involved in chaperone function (GroES/WD0308, DnaK/WD0928, and Hsp90/WD1277). The chaperone GroEL/WD0307, which putatively forms a complex with GroES/WD0308, is cotranscribed with GroES/WD0308 and shows similar down-regulation at later stages of the life cycle, but does not pass the significance threshold in the life cycle GLM (p = 0.15). Both GroES/WD0308 and GroEL/WD0307 were in the top 15 most abundant transcripts based on average TPM across all stages, confirming that chaperones are among the most highly expressed genes in *Wolbachia* (Bennuru *et al.* 2011; Darby *et al.* 2012, 2014). High basal expression of GroEL or other chaperone proteins has been suggested to be a compensatory mechanism for the accumulation of slightly deleterious nonsynonymous mutations in endosymbionts that arise because of their small population size and lack of recombination (Moran 1996; Fares *et al.* 2002). The differential expression of *Wolbachia* chaperones during the *D. melanogaster* life cycle that we have observed may result from different exposure to external sources of stress or different requirements for protein folding/stability between eggs and larvae *vs.* pupae and adults.

The second class, comprising the majority of up-regulated genes detected (57/80), shows increases in relative expression starting with the larval L1 or L2 stages carrying on into adulthood, with decreases at the larval L3 (12 hr) stage and increases at the white prepupal 2 and 3 d stages. Genes in this class are mostly unannotated, but include eight genes that code for proteins with membrane or secretion system function (WspB/WD0009, TerC/WD0194, SPFH domain/WD0482, type II secretion/WD0500, HlyD/WD0649, type I secretion/WD0770, VirB3/WD0859, Rhoptry surface protein related/WD1041) and four ANK-containing genes (WD0191, WD0385, WD0438, WD1213). ANK-containing genes from several bacterial species have been shown to be type IV secretion system effector molecules that have diverse effects on eukaryotic cells (Caturegli *et al.* 2000; Sisko *et al.* 2006; Lin *Et al*., 2007; Pan *Et al*., 2008; O’Brien *et al.* 2015). Thus, secretion of ANK-containing genes into the host cell may be enriched during early larval and mid-to-late pupal stages of *D. melanogaster* development. Up-regulation of components for secretion systems (type III) has been observed in pupal stages of other arthropod endosymbionts (Dale *et al.* 2002), suggesting that metamorphosis may be a general period that is enriched for up-regulation of secreted symbiont effector proteins involved in host interaction. *Wolbachia* genes up-regulated during pupal stages could play roles in bacterial proliferation or tissue-specific migration, since *Wolbachia* in *D. melanogaster* have previously been shown to increase in numbers during pupal stages of testis development (Clark *et al.* 2002). The presence of a homolog for the *Escherichia coli* transcriptional regulator DksA/WD1094 in this class also provides a potential mechanism to understand the common differential regulation of these genes (Paul *et al.* 2005; Costanzo *et al.* 2008).

A third class of 22 *Wolbachia* genes show up-regulation primarily in *D. melanogaster* adults, with higher expression in adult males relative to adult females at the same age (see more below). Most of the genes in this class also have no known function. However, three are ANK-containing genes (WD0291, WD0292, WD0438). Our observation of sex-biased expression of ANK-containing genes based on global gene expression profiles of *Wolbachia* in *D. melanogaster* extends results from targeted RT-PCR analysis showing sex-biased expression of ANK-containing genes in *Wolbachia* strains from other insects (Sinkins *et al.* 2005; Duron *et al.* 2007; Klasson *et al.* 2009; Papafotiou *et al.* 2011; Wang *et al.* 2014). Finally, we note that our qualitative classification of up-regulated genes in classes 2 and 3 is not mutually exclusive, and the existence of four genes (WD0438, WD1288, WD1289, and WD1290) with sex-biased expression that also show differential expression at larval or pupal stages suggests possible shared regulation of these classes.

### *Wolbachia* genes with sex-biased expression show age-dependent effects

**Figure 3:**
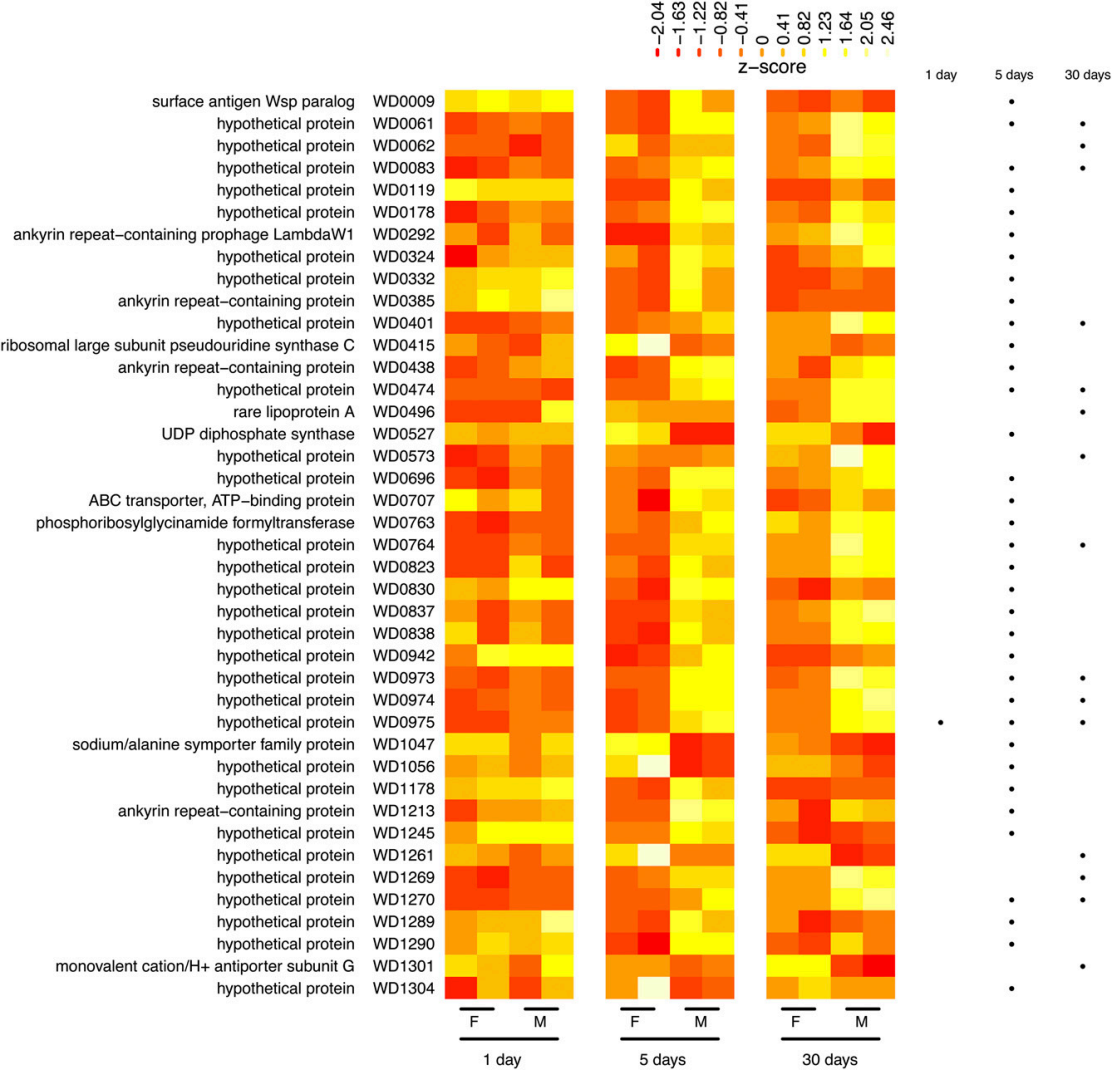
*Wolbachia* genes show age-dependent sex-biased expression. Row-normalized expression levels are visualized as a heatmap where each row represents a gene (ordered top-to-bottom by its position in the genome), and each cell represents the relative expression level for a particular sample in terms of Z-scores [observed transcripts per million (TPM) minus row mean TPM, divided by the standard deviation of TPMs for that row]. For each stage, two biological replicates are shown for each female (F) and male (M) sample as distinct columns. Values higher than row means are represented by yellow, and values lower than row means are represented by red. Gene names and identifiers are shown on the left. Dots on the right indicate if a gene is differentially expressed between males and females at 1, 5, or 30 d post eclosion, respectively. All 41 genes identified as differentially expressed in any of the three pairwise comparisons between males and females in ISO1 are shown here.

*Wolbachia* is known to cause a variety of sex-specific phenotypes in its hosts (Werren *et al.* 2008), including a form of embryonic mortality arising from matings between infected males and uninfected females known as cytoplasmic incompatibility (CI). The *w*Mel *Wolbachia* variant from *D. melanogaster* induces CI in the laboratory. However, this effect is partial and transient (Hoffmann *et al.* 1994; Yamada *et al.* 2007), and absent in field conditions (Hoffmann *et al.* 1998). To identify *Wolbachia* genes with sex-biased expression that might play a role in CI, we performed a more in-depth analysis of *Wolbachia* expression between males and females at matched ages. For this analysis, we used an exact testing framework (Robinson and Smyth 2008) because the GLM-based approach used for the complete life cycle is not the optimal method to use in a pairwise context. We identified a total of 41 genes that exhibited greater than 1.5-fold difference at an adjusted p-value cutoff of 0.01 in pairwise tests between male and female samples at either 1 d, 5 d, or 30 d post eclosion, respectively (Figure 3 and Table S2). Most sex-biased genes in this analysis were identified in one or both of the up-regulated classes in the life cycle GLM above (28/41, 68.3%), indicating that these complementary approaches identify a similar set of *Wolbachia* genes with detectable sex-biased expression in the modENCODE data set. Likewise, sex-biased genes comprise over one-third of differentially-expressed genes identified in the life cycle GLM (28/80, 35%), suggesting that sex-biased expression is a dominant component of the major differences in *Wolbachia* gene expression that can be observed across the *D. melanogaster* life cycle. Neither the GLM nor pairwise analysis revealed sex-biased expression in *D. melanogaster* for homologs of *Wolbachia* genes from *Culex pipiens* (WD0631, WD0632; WD0254, WD0255, WD0508, WD0622, WD0623, WD0626), which have recently been suggested to play a role in CI (Beckmann and Fallon 2013; Pinto *et al.* 2013).

The majority of sex-biased genes in the pairwise male-*vs.*-female analyses showed higher expression in males relative to females at matched stages, with only seven genes (rluC/WD0415, uppS/WD0527, sodium/alanine symporter/WD1047, WD1056, WD1261, cation antiporter subunit G/WD1301, WD1304) showing relatively higher expression in females at one or more time points. Many *Wolbachia* genes with male-biased expression are found in operons (Figure S4 and File S1). Additionally, most genes with sex-biased expression were identified at 5 d post eclosion (35/41), many of which maintained sex-biased expression until 30 d post eclosion. At 5 d post eclosion, whole-organism RNA-seq correctly predicted the presence (3/3) or lack (13/14) of sex-biased expression differences for 16/17 ANK-containing genes in a *w*Mel strain previously classified by RT-qPCR to have over a 1.5-fold difference in expression level between testes and ovaries of 2-d-old flies (Papafotiou *et al.* 2011) (the only exception being that WD0292 shows sex-biased expression in the RNA-seq data at 5 d that is not observed in the RT-qPCR at 2 d). The general lack of sex-biased expression at 1 d post eclosion inferred from RNA-seq is also supported by RT-qPCR results (Figure S2): the five up-regulated genes we tested are all sex-biased at 5 d but not 1 d post eclosion in the RNA-seq data (Figure 3), and none of these genes show sex-biased expression at 1 d post eclosion in our RT-qPCR data.

Our finding that *Wolbachia* genes with sex-biased expression are typically up-regulated at 5 d post eclosion is puzzling considering previous work showing a decline in the strength of CI in *D. melanogaster* males at 1 *vs.* 5 d post eclosion (Reynolds and Hoffmann 2002; Reynolds *et al.* 2003; Yamada *et al.* 2007). Given that the CI phenotype can vary in *D. melanogaster* (Hoffmann *et al.* 1998), it is possible that CI was not expressed in the ISO1 samples used for the modENCODE time course and, thus, *Wolbachia* genes that are up-regulated as males age have nothing to do with CI. If so, these results may imply that *Wolbachia* responds to or affects other sexually dimorphic host phenotypes that vary with age. If these genes are in fact involved in CI, however, the observed pattern of sex-biased genes being up-regulated in older males would be compatible with these *Wolbachia* genes playing a role in attenuating the modification of *D. melanogaster* sperm that leads to embryonic lethality in incompatible crosses (Poinsot *et al.* 2003). Alternatively, if the host is responsible for reducing the effects of *Wolbachia* on the sperm of older males, up-regulation of *Wolbachia* genes in older males could represent an attempt by *Wolbachia* to compensate against host attenuation and hence indicate these genes play a role in promoting CI.

### wMel genes with dynamic expression in *D. melanogaster* are conserved in other *Wolbachia* strains

To understand if candidate genes identified on the basis of differential expression in *D. melanogaster* might interact more broadly with other hosts, we asked whether *w*Mel genes that show stage- and sex-specific expression are conserved in other divergent *Wolbachia* strains. For this analysis, we used complete *Wolbachia* genome sequences from *w*Ri (an arthropod supergroup A strain from *D. simulans*), *w*Pip-Pel (an arthropod supergroup B strain from *Culex quinquefasciatus*) and *w*Bm (a nematode supergroup D strain from *Brugia malayi*) (Klasson *et al.* 2008, 2009; Foster *et al.* 2005). We identified and clustered homologs in all genomes analyzed, and reconstructed homology groups that included *w*Mel homologs for 86 of 93 genes that show either stage- or sex-specific expression (seven dynamically-expressed *w*Mel genes were too small to pass BLAST filtering cutoffs). Only three of the 86 dynamically-expressed genes in homology groups (3.5%) were restricted to the *w*Mel genome, whereas 30 genes (34.9%) had homologs in *Wolbachia* genomes from other arthropods, and a further 53 genes (61.6%) also had homologs in *Wolbachia* genomes from nematodes. The phylogenetic distribution of dynamically-expressed genes does not differ from genome-wide expectations (χ^2^ = 4.82, p = 0.09, d.f. = 2). These results indicate that the majority of genes identified as dynamically expressed in *D. melanogaster* are core components of the *Wolbachia* gene repertoire and are not unusual in their degree of conservation. Nevertheless, many dynamically-expressed candidate genes are arthropod-specific but only a few are *D. melanogaster*-specific, as might be expected for candidate host-interaction genes in a facultative endosymbiont that can switch arthropod hosts by horizontal transfer. Arthropod-specific dynamically-expressed genes include several ANK-containing genes (WD0191, WD0636, WD1213) and membrane/secretion system genes (ABC transporter/WD0455, SPFH domain/WD0482, type II secretion/WD0500, sodium/alanine symporter/WD1047, ClpA/WD1237), emphasizing the importance of intercellular communication in explaining how *Wolbachia* forms facultative symbioses with its arthropod hosts.

### Expression of *Wolbachia* genes previously implicated in host-microbe interaction

**Figure 4:**
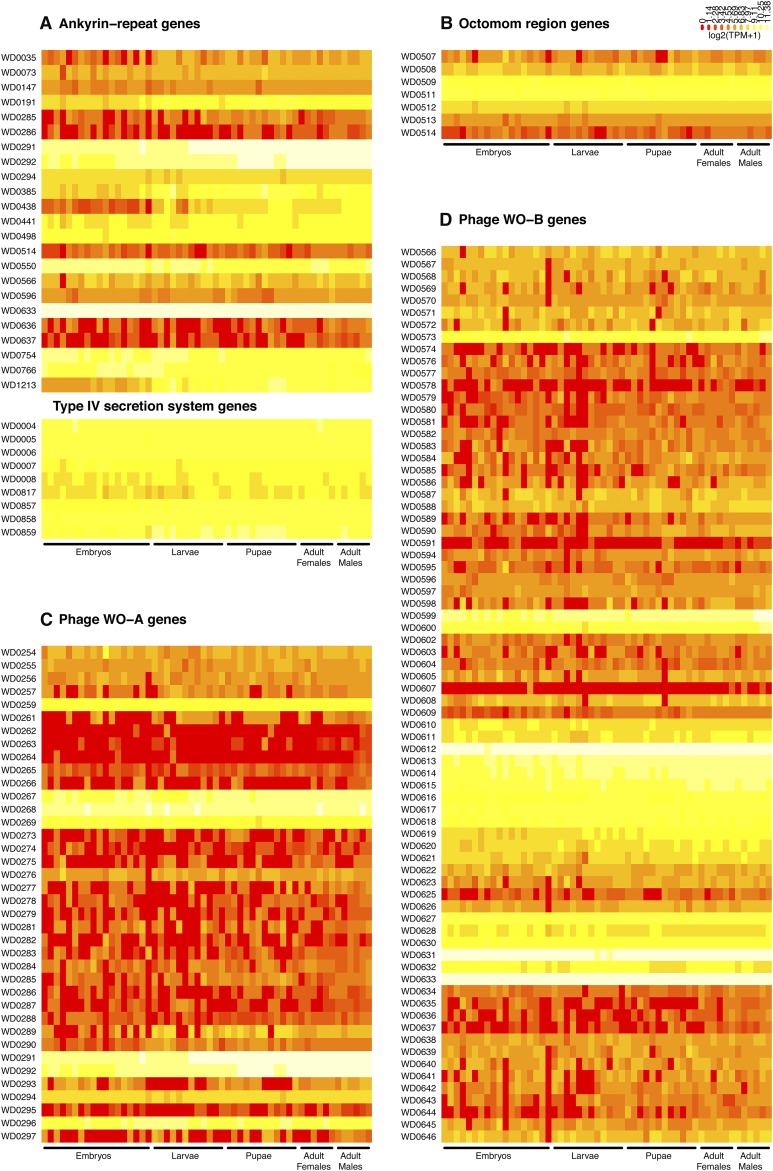
Expression profiles of *Wolbachia* genes previously implicated in host-microbe interactions. (A) ANK-containing and type IV secretion system genes. (B) Octomom genes. (C) Prophage WO-A genes. (D) Prophage WO-B genes. The 23 ANK-containing genes in panel (A) are distributed throughout the *Wolbachia* genome. The nine type IV secretion system genes are found in three different genomic intervals. The Octomom, prophage WO-A, and prophage WO-B regions are each from single intervals in the *Wolbachia* genome. The Octomom region only contains seven genes, since WD0510 is not included in the Ensembl annotation for *Wolbachia w*Mel. Phage coordinates are from Metcalf *et al.* (2014). Expression levels are not row-normalized and are visualized as a heatmap where each row represents a gene (ordered top-to-bottom by its position in the genome) and each cell represents expression in units of observed transcripts per million (TPM). A pseudocount of one was added to each gene’s TPM before transforming to log_2_ scale. Values with higher levels of expression are represented by yellow, and values with lower levels of expression are represented by red. All panels are on the same heatmap color scale. Gene names and identifiers are shown on the left. All stages including those that lack biological replicates in the modENCODE time course are shown here.

In addition to identifying new candidates for mediating host-microbe interaction on the basis of their stage- or sex-specific differential expression, we also investigated expression levels of *Wolbachia* genes previously suggested to be candidates for mediating interaction with *D. melanogaster*. The most widely hypothesized set of candidates for host-microbe interaction are the 23 ANK-containing genes that are possible type IV secretion system effectors in *Wolbachia* (Wu *et al.* 2004; Iturbe-Ormaetxe *et al.* 2005; Papafotiou *et al.* 2011; Siozios *et al.* 2013), which the modENCODE data show are expressed at widely different levels in *D. melanogaster* (Figure 4A). The five most weakly-expressed ANK-containing genes (WD0285, WD0286, WD0514, WD0636, WD0637) are found in the Octomom and prophage regions, and are the same five genes that Papafotiou *et al.* (2011) found were too weakly expressed to obtain reliable RT-qPCR data in adult gonads. Thirteen ANK-containing genes are highly expressed (WD0191, WD0291, WD0292, WD0294, WD0385, WD0438, WD0441, WD0498, WD0550, WD0633, WD0754, WD0766, WD1213), which include the majority of differentially expressed ANK-containing genes identified in this study or by Papafotiou *et al.* (2011) (WD0191, WD0291, WD0292, WD0294, WD0385, WD0438, WD0550, WD0636, WD1213). The nine genes that make a complete type IV secretion system in *w*Mel are all highly expressed in all *D. melanogaster* life cycle stages, including the virB8 paralog (WD0817), which is not a part of the two genomic clusters that contain the remaining eight type IV secretion system genes. These results together support a model where a functionally competent type IV *Wolbachia* secretion system is expressed throughout the *D. melanogaster* life cycle, with both constitutive and regulated secretion of subsets of ANK-containing effectors.

The Octomom region is part of the accessory genome of *Wolbachia* (Iturbe-Ormaetxe *et al.* 2005; Chrostek *et al.* 2013) and contains eight genes whose copy number controls *Wolbachia* growth and pathogenesis (Chrostek and Teixeira 2015). In the *w*Mel variant, where Octomom is present in one copy, all genes in this region (WD0507–WD0514) are expressed at relatively constant levels across the life cycle (Figure 4B). None of these genes show a significant change in gene expression during host development in the life cycle GLM. However, different Octomom genes do vary considerably in their expression levels relative to each other, with the most highly expressed genes being found in the middle of this interval (WD0509–WD0512). Given that two Octomom genes with possible regulatory (the helix-turn-helix-containing gene WD0508) or effector (the ANK-containing gene WD0514) function are relatively weakly expressed in the nonpathogenic *w*Mel variant, it is possible that overexpression of one or both of these genes may be responsible for the pathogenic phenotype when Octomom is amplified in *w*MelPop (Chrostek and Teixeira 2015).

Unlike obligate endosymbionts with streamlined genomes, prophages are often present in *Wolbachia* from arthropod hosts and have been suggested to directly or indirectly influence *Wolbachia*-host interactions (Kent and Bordenstein 2010; Metcalf *et al.* 2014). Two major prophage regions are present in the *w*Mel genome, called WO-A and WO-B, both of which have undergone degeneration and rearrangement since insertion (Wu *et al.* 2004; Kent *et al.* 2011). There is no clear evidence that the WO-A and WO-B prophages from *w*Mel can enter a lytic phase as they can in other arthropods (Masui *et al.* 2001; Fujii *et al.* 2004; Bordenstein *et al.* 2006; Sanogo and Dobson 2006). However, phage-like particles have been reported in extracts of *D. melanogaster* strains infected with *w*Mel (Gavotte *et al.* 2004). Consistent with previous results from *w*MelPop-CLA (Darby *et al.* 2014) and prophages in *Salmonella enterica* (Perkins *et al.* 2009), expression levels of most genes in the WO-A and WO-B prophage regions are typically very low across the entire *D. melanogaster* life cycle (Figure 4, C and D), and define the largest segments of the *w*Mel genome with consecutive lowly-expressed genes. The most conspicuous exception to this pattern is the 21 gene interval in WO-B (WD0611–WD0634) that contains genes laterally-transferred between *Wolbachia* and the *Rickettsia* endosymbiont of the tick *Ixodes scapularis* (WD0612–WD0621) (Ishmael *et al.* 2009; Gillespie *et al.* 2012), a region not typically present in WO prophage from other *Wolbachia* strains (Kent *et al.* 2011). In addition, two very abundant currently-unannotated antisense ncRNA transcripts can be detected overlapping the major capsid genes of both WO-A (WD0274) and WO-B (WD0604) (Figure S5), which may play a role in the regulation of prophage genes.

Most prophage-encoded structural genes are expressed at low levels, with only genes in the tail (WD0567–WD0574) and base-plate (WD0638–WD0644) regions of WO-B being expressed at appreciable levels. Likewise, most nonstructural prophage-encoded genes previously suggested to be candidates for host interaction (VrlC.2/WD0579, VrlC.1/WD0580, Patatin/WD0565, DNA methylases WD0263 and WD0594) (Kent and Bordenstein 2010) are expressed at low levels. Intriguingly, each prophage region contains a highly-expressed operon (WD0267–WD0269 in WO-A and WD0599–WD0600 in WO-B) that encodes homologs of the *E. coli* RelE toxin (WD0269 and WD0600) (Figure 4, C and D). RelE is a stress-inducible cytotoxic translational repressor that is counteracted by the antitoxin RelB, a small protein, the gene of which is cotranscribed in the same operon as RelE (Christensen *et al.* 2001; Pedersen *et al.* 2003; Yamaguchi *et al.* 2011). The genes adjacent to the RelE homologs in WO-A and WO-B (WD0267, WD0268 and WD0599) are also cotranscribed and encode small peptides, and thus could be acting as antitoxins. In fact, WD0268–WD0269 and WD0599–WD0600 have been computationally predicted to be toxin-antitoxin pairs (Shao *et al.* 2011), and toxin-antitoxin pairs have been previously reported in other cryptic prophages (Van Melderen and Saavedra De Bast 2009). RelE-containing gene clusters are also found in similar positions (between the terminase and portal genes that form the phage head) in divergent prophages from *D. simulans* (WOriA) and *Nasonia vitripinnis* (WOVitA2) (Kent *et al.* 2011), further indicating that they may play some conserved functional role such as stabilizing the *Wolbachia* prophage genomic regions by preventing large-scale deletions (Van Melderen and Saavedra De Bast 2009). Low expression levels of phage structural genes together with highly-expressed putative toxin-antitoxin pairs suggests that prophages in *w*Mel are maintained in the lysogenic state by self-preservation.

### Conclusions and Future Directions

We have shown that the ISO1 reference strain used by the modENCODE project is infected with *Wolbachia*, and used this fortuitous observation to study the global expression dynamics of a facultative endosymbiont over the life cycle of the model insect species *D. melanogaster*. Our work represents the most comprehensive gene expression profiling of an endosymbiotic bacteria in its native host context to date. We have established that most *Wolbachia* genes are expressed in all *D. melanogaster* life cycle stages, but that major changes in expression levels of *Wolbachia* genes are rare when studied simultaneously across all *D. melanogaster* tissues. It is important to emphasize, however, that the modENCODE total RNA-seq libraries were made from whole animals, and thus any tissue-specific *Wolbachia* gene expression differences that may exist cannot be detected in these data, nor can sex-specific differences in nonadult stages. Nevertheless, we identify a set of 93 *Wolbachia w*Mel genes that show robust stage- or sex-specific differential expression at the whole-fly level, many of which share common expression dynamics and therefore may be coregulated. These genes provide many new candidate genes for understanding, and possibly manipulating, the genetic basis of how *Wolbachia* interacts with arthropod hosts. Importantly, we also provide the first detailed insight into the developmental dynamics of *Wolbachia* gene expression in an insect host, which suggests that the larval and pupal stages [where *Wolbachia* have been detected cytologically (Clark *et al.* 2002, 2003, 2005)] merit further study to understand how *Wolbachia* manipulates host biology to maintain persistent infections and affect transmission.

Future studies can leverage our finding that the modENCODE total RNA-seq dataset contains a nearly-complete *Wolbachia* transcriptome to functionally annotate the transcriptional landscape of the *Wolbachia* genome. Currently, only protein-coding regions and a small number of ncRNAs are included in the *w*Mel genome annotation (Wu *et al.* 2004), and recent work has identified a handful of additional *Wolbachia* ncRNAs (Mayoral *et al.* 2014; Woolfit *et al.* 2015). The strand-specific total RNA-seq data from modENCODE can now be used to generate high quality transcript models to annotate 5′- and 3′-untranslated regions of protein-coding genes, delimit operons, and identify new ncRNA genes in *Wolbachia* (see examples in Figure S4 and Figure S5). The possibility of a more comprehensive annotation of ncRNAs in *Wolbachia* is particularly exciting given recent work suggesting that ncRNAs provide an important layer of posttranscriptional regulation to modulate protein expression levels in the *Buchnera* endosymbiont of aphids (Hansen and Degnan 2014). Together with other recently published transcriptomic data (Darby *et al.* 2014; Mayoral *et al.* 2014; Woolfit *et al.* 2015), the necessary materials are now available to undertake a systematic reannotation of the *Wolbachia w*Mel genome in order to support basic and applied research on this important model organism.

## 

## Supplementary Material

Supporting Information
