# Supplementary material for: Dynamics of Wolbachia pipientis Gene Expression Across the Drosophila melanogaster Life Cycle
Source: G3 (Bethesda). 2015 Oct 23;5(12):2843–56. doi: 10.1534/g3.115.021931 (PMC4683655; doi:10.1534/g3.115.021931)
Supplement: Supporting Information [file supp_5_12_2843__index.html]

Dynamics of Wolbachia pipientis Gene Expression Across the Drosophila melanogaster Life Cycle — Supporting Information 

# Dynamics of *Wolbachia pipientis* Gene Expression Across the *Drosophila melanogaster* Life Cycle

## Supporting Information for Gutzwiller *et al.*, 2015

**Files in this Data Supplement:**

- Supporting Information - Contains Figures S1-S5, File S1, and legends for Tables S1-S4. (.pdf, 101 KB)
- Figure S3 - Clustering analysis of *Wolbachia* gene expression in the modENCODE life cycle time course. (.pdf, 344 KB)
- Figure S4 - *Wolbachia* genes with sex-biased expression are often found in operons. (.pdf, 299 KB)
- Figure S5 - Putative anti-sense noncoding RNAs in the *Wolbachia* WO-A and WO-B regions. (.pdf, 75 KB)
- File S1 - Supporting Results and Discussion. (.pdf, 101 KB)
- Figure S1 - *Wolbachia* gene expression levels are highly-correlated across biological replicates and life cycle stages. (.pdf, 85 KB)
- Figure S2 - Confirmation of stably- and differentially-expressed genes by RT-qPCR. (.pdf, 209 KB)
- Table S1 - Summary of RNA-seq datasets. (.tsv, 6 KB)
- Table S2 - Expression levels, conservation status and metadata for *Wolbachia* genes. (.tsv, 2,002 KB)
- Table S3 - Results of the RT-qPCR GLM analysis. (.tsv, 2 KB)
- Table S4 - RT-qPCR primers. (.tsv, 1 KB)
