# Supplementary material for: Dynamics of Wolbachia pipientis Gene Expression Across the Drosophila melanogaster Life Cycle
Source: G3 (Bethesda). 2015 Oct 23;5(12):2843–56. doi: 10.1534/g3.115.021931 (PMC4683655; doi:10.1534/g3.115.021931)
Supplement: Supporting Information [file supp_g3.115.021931_FigureS3.pdf]

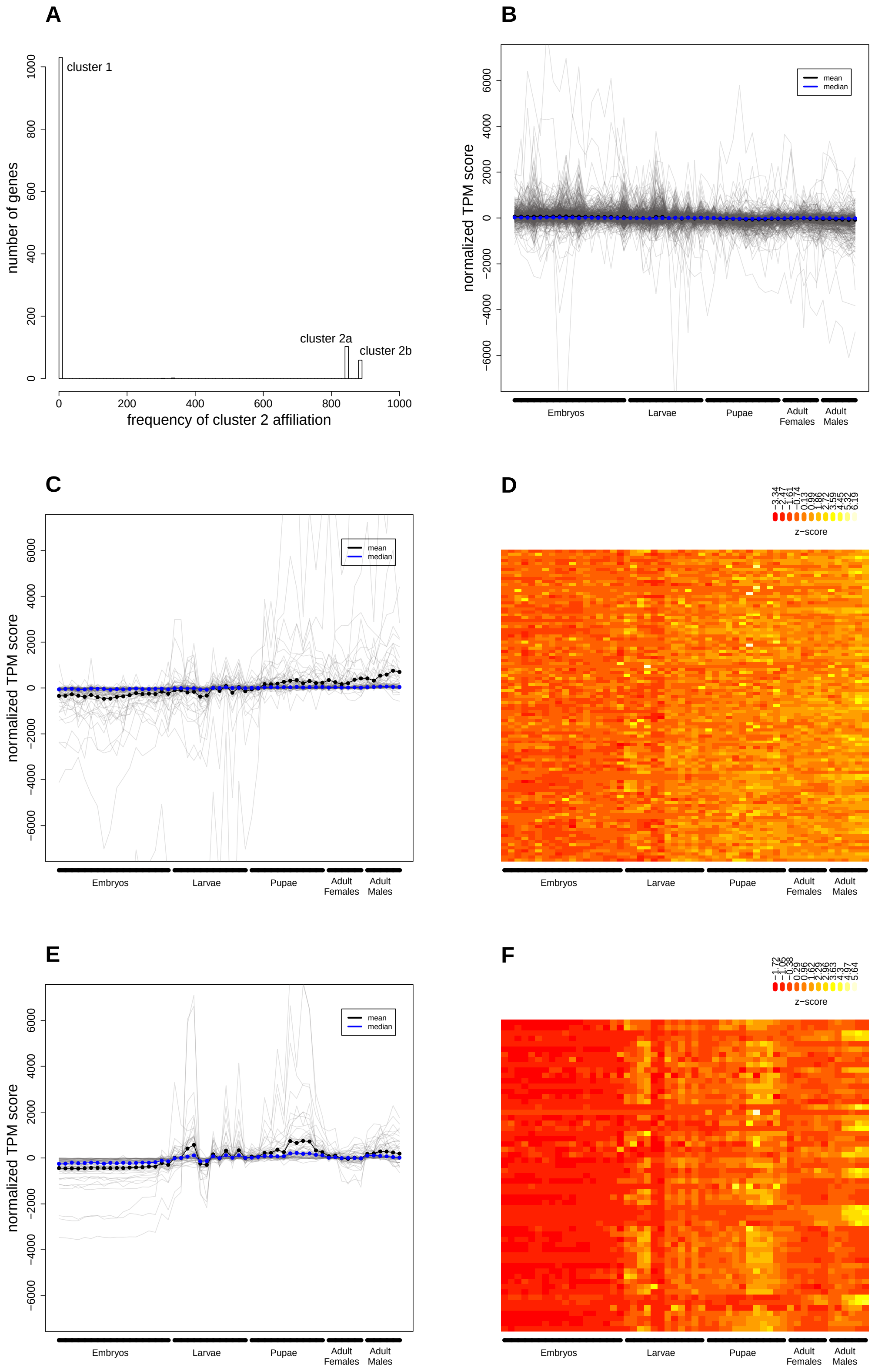
A
B
C
D
E
F
cluster 1
cluster 2a
cluster 2b
number of genes
frequency of cluster 2 affiliation
normalized TPM score
mean
median
z−score
Embryos
Larvae
Pupae
Adult Females
Adult Males

Figure S3. Clustering analysis of *Wolbachia* gene expression in the modENCODE life cycle time course.

(A) Histogram showing the number of independent clustering runs (out of a total of 1000 runs) using MBcluster.seq (Si *et al.*, 2014) that a gene was affiliated with the variable gene cluster 2. Three distinct peaks were observed, one for stably-expressed genes (cluster 1, n=1033), and two peaks for variable genes that we denote cluster 2a (n=103) and cluster 2b (n=59). (B) Normalized expression profiles for genes in Cluster 1. (C) Normalized expression profiles for genes in Cluster 2a. (D) Heat map of row-normalized expression levels for genes in Cluster 2a. (E) Normalized expression profiles for genes in Cluster 2b. (F) Heat map of row-normalized expression levels for genes in Cluster 2b. For panels (B), (C) and (E), TPMs for each gene at each stage are normalized (shown in grey) by subtracting the mean TPM for that gene across different life cycle stages (shown in black). The mean and median of normalized expression levels for all genes at each stage are show for each cluster in black and blue, respectively. For panels (D) and (F), row-normalized expression levels are visualized as a heatmap where each row represents a gene (ordered top-to-bottom by its position in the genome), and each cell represents the relative expression level for a particular sample in terms of Z-scores (observed TPM minus row mean TPM, divided by the standard deviation of TPMs for that row). Values higher than row means are represented by yellow, and values lower than row means are represented by red. Note that the heatmap color scale differs in panels (D) and (F).
