# Supplementary material for: Dynamics of Wolbachia pipientis Gene Expression Across the Drosophila melanogaster Life Cycle
Source: G3 (Bethesda). 2015 Oct 23;5(12):2843–56. doi: 10.1534/g3.115.021931 (PMC4683655; doi:10.1534/g3.115.021931)
Supplement: Supporting Information [file supp_g3.115.021931_FigureS4.pdf]

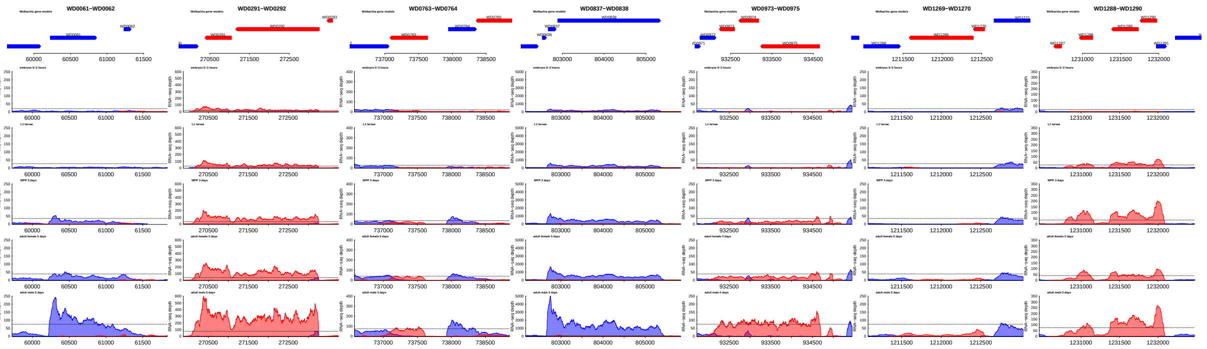

Figure S4. *Wolbachia* genes with sex-biased expression are often found in operons.

Wiggle plots of *Wolbachia* expression levels for seven clusters of *Wolbachia* genes with sex-biased expression. Gene models and RNA-seq coverage for each stage are shown for the forward and reverse strands in blue and red, respectively. RNA-seq plots are shown on the same absolute y-axis scale. To provide an internal normalization factor for comparison across samples, mean coverage of the stably-expressed Wsp/WD1063 gene (not shown in this interval) divided by twenty is depicted by the dashed line in each panel. Six out of seven clusters (WD0061–WD0062, WD0291–WD0292, WD0837–WD0838, WD0973–WD0975, WD1269–WD1270 and WD1288–WD1290) were confirmed as operons based on contiguous mapping of RNA-seq reads. The third cluster depicted contains two divergently transcribed genes (WD0763–WD0764) that are not co-transcribed as an operon.
