# Supplementary material for: Dynamics of Wolbachia pipientis Gene Expression Across the Drosophila melanogaster Life Cycle
Source: G3 (Bethesda). 2015 Oct 23;5(12):2843–56. doi: 10.1534/g3.115.021931 (PMC4683655; doi:10.1534/g3.115.021931)
Supplement: Supporting Information [file supp_g3.115.021931_FigureS5.pdf]

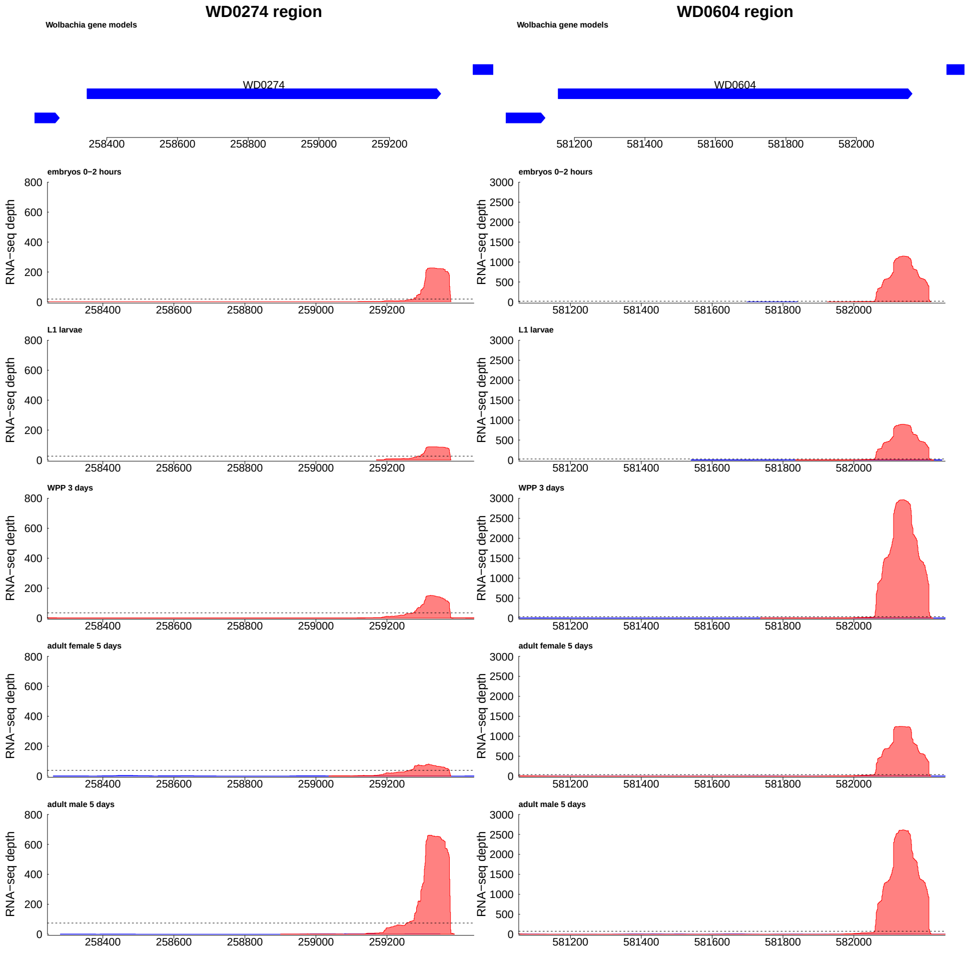

Figure S5. Putative anti-sense noncoding RNAs in the *Wolbachia* WO-A and WO-B regions.

Wiggle plots of *Wolbachia* expression levels for two highly expressed putative anti-sense noncoding RNA genes that overlap the 3' ends of the major phage capsid genes of both WO-A (WD0274) and WO-B (WD0604). Gene models and RNA-seq coverage for each stage are shown for the forward and reverse strands in blue and red, respectively. RNA-seq plots are shown on the same absolute y-axis scale. To provide an internal normalization factor for comparison across samples, mean coverage of the stably-expressed Wsp/WD1063 gene (not shown in this interval) divided by twenty is depicted by the dashed line in each panel. These transcribed regions are the most highly expressed sequences in both of the WO-A and WO-B regions, and are found in conserved locations of paralogs with divergent sequences.
