# Supplementary material for: Dynamics of Wolbachia pipientis Gene Expression Across the Drosophila melanogaster Life Cycle
Source: G3 (Bethesda). 2015 Oct 23;5(12):2843–56. doi: 10.1534/g3.115.021931 (PMC4683655; doi:10.1534/g3.115.021931)
Supplement: Supporting Information [file supp_g3.115.021931_FileS1.pdf]

File S1.
Supporting Results and Discussion.

Provenance and infection status of ISO1 sub-strains

We investigated the cause of the discrepancy of *Wolbachia* sequences in the Berkeley *Drosophila* Genome Project (BDGP) and Bloomington *Drosophila* Stock Center (BDSC) ISO1 genomic data by first establishing the provenance of these lineages. As documented in the BDSC database, the BDSC ISO1 sub-strain was donated directly to the stock center by Jim Kennison in 1994. The ISO1 sub-strain used by the BDGP was obtained from Gerry Rubin's lab in the late 1990's (Roger Hoskins, personal communication). The Rubin lab ISO1 sub-strain was obtained in the mid-1990s from Jim Kennison *via* at least one intermediate lab (Todd Laverty, personal communication). Thus, in contrast to naive assumptions, the ISO1 sub-strain at the Bloomington *Drosophila* Stock Center is neither a direct descendant nor progenitor of the ISO1 sub-strain used in the *D. melanogaster* genome project, and these two sub-strains have had independent trajectories since at least 1994.

The presence of *Wolbachia* sequences in our genomic data, but not in BDGP genomic data, could be explained in a number of ways. For example, the BDGP ISO1 sub-strain could have lost its infection, or *Wolbachia* sequences could have been eliminated during nuclear enrichment steps that were used to reduce mitochondrial DNA in the original Sanger sequencing libraries (Roger Hoskins, personal communication) (Adams *et al.*, 2000). Alternatively, the ISO1 sub-strain could alternatively have acquired a *Wolbachia* infection in the BDSC where many stocks are known to be infected (Clark *et al.*, 2005), although this is less likely because horizontal transfer of *Wolbachia* has not been observed in *D. melanogaster* whereas spontaneous loss is relatively common (Hoffmann *et al.*, 1998; Richardson *et al.*, 2012).

To resolve when and where the BDSC ISO1 sub-strains lost (or acquired) its *Wolbachia* infection, we obtained ISO1 sub-strains from three additional sources: (i) the original sub-strain, maintained continuously by Jim Kennison since its synthesis in 1986; (ii) the sub-strain used for the *Drosophila* genome project, maintained by the BDGP since the late 1990's; and (iii) a sub-strain maintained by Gerry Rubin's lab since the mid-1990's, which was the progenitor of the BDGP sub-strain. The Rubin lab sub-strain was never treated for a *Wolbachia* infection using antibiotics (Gerry Rubin, personal communication). We performed diagnostic PCR for

Wolbachia on all four ISO1 sub-strains before and after tetracycline treatment. We found that the ISO1 sub-strains from Jim Kennison's lab and the BDSC were infected with *Wolbachia*, but those from the Rubin lab and the BDGP were not (data not shown). Amplification of *Wolbachia* sequences was not observed in flies after antibiotic treatment, indicating that the source of *Wolbachia* DNA sequences is not due to nuclear integration, which occurs rarely in *D. melanogaster* (Huang *et al.*, 2014).

We conclude that the original ISO1 strain synthesized by Kennison was infected with *Wolbachia*, that an infected ISO1 sub-strain was donated by Kennison to the BDSC in 1994, and that an ISO1 sub-strain that was cured of (or spontaneously lost) its infection was obtained by the Rubin lab and donated to the BDGP. These results also show that, in addition to a high proportion of lab stocks carrying *Wolbachia* (Clark *et al.*, 2005), sub-lines of the same *D. melanogaster* stock may vary in their *Wolbachia* infection status.

Characterization of *Wolbachia* transcriptomic data in modENCODE libraries

To provide an overview of the *Wolbachia* transcriptomic data present in modENCODE libraries, we counted reads and estimated expression levels in transcripts per million (TPM) for each of the 1195 characterized protein-coding genes in the *Wolbachia* genome in each of the modENCODE RNA-seq samples (Table S1). At least 948 (79.3%) *Wolbachia* genes were expressed (defined as having non-zero TPM estimates) across all stages and replicates (Table S1). Using the more stringent definition of ≥ 2 mapped reads per gene used by Darby *et al.* (Darby *et al.*, 2014), more *Wolbachia* genes can be detected as being expressed in each sample from the modENCODE whole-organism RNA-seq data set ($>$856 genes, $>$71.6% of total) than could be detected by these authors using combined transcriptomic and proteomic profiling of *Wolbachia* *w*MelPop-CLA expression in cell culture (66.8%). The two samples with the lowest percentage of expressed genes were also among those with the fewest total reads, and both had many more expressed genes in the other biological replicate from the same stage (Embryos 22-24 hr: 948 vs 1111, Embryos 12-14 hr: 994 vs 1112, Table S1), indicating these samples may be outliers with respect to the actual number of genes expressed because of low sequencing coverage. The median number of genes expressed (non-zero TPM) across all samples is 1110, indicating that the majority of the *Wolbachia* genome is transcriptionally active throughout the *D. melanogaster* life cycle.

Additionally, we measured the correlation of expression levels (measured in TPMs) across all *Wolbachia* genes between all pairs of samples in the modENCODE total RNA-seq time course (Figure S1). Correlation among biological replicates of the same stage is very high ($r \geq 0.94$) and highly significant ($p < 2.2e - 16$). The lowest correlation among biological replicates is observed for three stages from the third larval instar (L3 dark blue gut, L3 light blue gut and L3 clear gut), which show higher similarity between stages from the same replicate series than they do between biological replicates from the same stage. Correlation of expression levels is also reasonably high and highly significant among all life-cycle stages ($r > 0.61$, $p < 2.2e - 16$). Overall, these results indicate that reproducibility of expression levels among biological replicates in the modENCODE data is high enough and the magnitude of expression differences among stages is low enough to perform differential expression analysis, and that the global pattern of *Wolbachia* gene expression does not change dramatically across the *D. melanogaster* life cycle when assayed at the level of the whole organism.

Quantitative analysis of *Wolbachia* RT-qPCR gene expression data

We validated the major pattern of *Wolbachia* gene expression dynamics between embryos and adults based on RNA-seq by performing reverse-transcriptase real-time quantitative PCR (RT-qPCR) for a sample of 10 genes in the BDSC ISO1 sub-strain. To assess if our findings were restricted to only one specific host strain, we also performed RT-qPCR for the same genes as well as in a second *D. melanogaster* strain (DrosDel w1118) that carries a *w*Mel genotype very closely related to the one infecting ISO1 (see Figure 1A) (Chrostek *et al.*, 2013). Three *Wolbachia* genes that were not identified as differentially expressed were chosen as reference genes (WD1043, Wsp/WD1063, petB/WD1071) on the basis of a low fold-change and low coefficient of variation across the RNA-seq time course. One of these (WD1063) is Wsp, the most highly expressed gene in the *w*Mel genome that has been used previously as a control for RT-qPCR-based expression analysis in *Wolbachia* (Papafotiou *et al.*, 2011). We chose five genes that were up-regulated after embryogenesis (WspB/WD0009, WD0061, WD0830, WD0837, WD1289) and two genes that were down-regulated after embryogenesis (groES/WD0308, Hsp90/WD1277) to validate results of the GLM-based RNA-seq differential expression analysis. We performed RT-qPCR on 16-18 hr old embryos, 1-day post-eclosion males, and 1-day post-eclosion virgin females. We measured relative expression levels normalized to the average expression in embryonic samples of the three stably-expressed genes.

As shown in Figure S2, RT-qPCR in both ISO1 and DrosDel w1118 broadly confirmed that genes predicted to be stably-expressed based on RNA-seq do not change substantially in relative expression from embryonic to adult stages, whereas both up-regulated and down-regulated genes do, and in the same direction and relative magnitude as predicted by the RNA-seq analysis. Generalized linear modelling of RT-qPCR expression levels for ISO1 and DrosDel w1118 separately revealed more genes with significant differences between embryos and adults in DrosDel w1118 (upregulated: 5/5 validated; down-regulated: 2/2 validated) than in ISO1 (up-regulated: 4/5 validated; down-regulated: 1/2 validated) (Table S3). We also detected slight but significant differences in RT-qPCR expression between embryos and adults for two genes in the "stable" class in DrosDel w1118 (Wsp/WD1063, petB/WD1071), which are not observed in ISO1.

To increase overall power and to separate gene-specific from strain-specific effects, we analysed RT-qPCR data for both strains together in a single GLM (Table S3). This joint analysis revealed that all seven genes predicted to be up- or down-regulated based on RNA-seq in ISO1 were confirmed as differentially expressed between embryos and adults using RT-qPCR. Differential expression was observed for all genes in both sexes with the exception of Hsp90/WD1277, which only showed significant differences between embryos and males. Genes predicted to be stably-expressed are also confirmed, with the exception of WD1043 which shows a slight, but marginally significant up-regulation in males versus embryos. Overall, we conclude that RT-qPCR results validate the *Wolbachia* gene expression dynamics inferred from whole-organism RNA-seq in *D. melanogaster*, and that gene expression information from the ISO1 RNA-seq time course can likely be extrapolated to other strains carrying *w*Mel-like *Wolbachia* infections.

Probabilistic clustering of *Wolbachia* gene expression across the *D. melanogaster* life cycle

Probabilistic clustering (Si *et al.*, 2014) on the entire modENCODE RNA-seq time course (including stages without replicates) revealed that the majority of *Wolbachia* genes (1033, 86.4%) show a pattern of relatively stable expression levels across the life cycle (Figure S3, panels A and B). These results demonstrate that only a small subset of *Wolbachia* genes show robust differences in expression across the modENCODE life-cycle time course, supporting similar conclusions based on the the life-cycle GLM differential expression analysis. Genes identified as dy-

namically expressed by clustering include the vast majority of genes identified as differentially expressed in the life-cycle GLM (74/80, 92.5%). However, six genes (GroES/WD0308, ABC transporter/WD0455, succinate dehydrogenase subunit/WD0727, WD0804, DnaK/WD0928, Hsp90/WD1277) identified as differentially expressed in the life-cycle GLM were not found in cluster 2 and are instead merged together with the stably-expressed genes in cluster 1, most of which (with the exception of succinate dehydrogenase subunit/WD0727) are all in the small, down-regulated class of differentially-expressed genes.

Closer inspection of the number of runs in which a gene was assigned to cluster 2 also revealed two distinct sub-clusters (Figure S3, panel A), both of which show up-regulation after embryogenesis. The first, which we call cluster 2a, shows modest up-regulation after embryogenesis with the characteristic dip in expression at larval L3 (12 hrs) for a set of 103 genes, and includes a substantial proportion (22/75, 29.7%) of the up-regulated genes identified in the life-cycle GLM (Figure S3, panel C). The second, cluster 2b, shows stronger up-regulation for 59 genes and the dip in expression at larval L3 (12 hrs), including the majority of both up-regulated classes identified in the life-cycle GLM (52/75, 70.3%) (Figure S3, panels E and F).

Wolbachia genes with male-biased expression are often found in operons

Many *Wolbachia* male-biased genes identified in either the life-cycle GLM or male-vs-female pairwise analyses are found consecutively in the genome in small clusters (WD0061–WD0062, WD0291–WD0292, WD0763–WD0764, WD0837–WD0838, WD0973–WD0975, WD1269–WD1270 and WD1288–WD1290), suggesting that they might be co-transcribed as operons (see example in Figure 1B). Visual inspection confirmed that six out of seven of these clusters (WD0061–WD0062, WD0291–WD0292, WD0837–WD0838, WD0973–WD0975, WD1269–WD1270 and WD1288–WD1290) are on the same strand and co-transcribed as operons based on contiguous mapping of RNA-seq reads between genes (Figure S4). The remaining cluster (WD0763–WD0764) corresponds to two divergently-transcribed genes whose co-regulation may represent transcription from a common promoter region. While operon structure is predicted to be common in the *Wolbachia* genome (Pertea *et al.*, 2009; Dehal *et al.*, 2010; Taboada *et al.*, 2012; Mao *et al.*, 2014), the detection of sex-biased expression for individual genes in the same operon mutually reinforces that these genes are truly differentially expressed, and suggests common functionality of the genes in these as-yet uncharacterized loci.

References

Adams, M. D., S. E. Celniker, R. A. Holt, C. A. Evans, J. D. Gocayne, P. G. Amanatides, S. E. Scherer, P. W. Li, R. A. Hoskins, R. F. Galle, R. A. George, S. E. Lewis, S. Richards, M. Ashburner, S. N. Henderson, G. G. Sutton, J. R. Wortman, M. D. Yandell, Q. Zhang, L. X. Chen, R. C. Brandon, Y.-H. C. Rogers, R. G. Blazej, M. Champe, B. D. Pfeiffer, K. H. Wan, C. Doyle, E. G. Baxter, G. Helt, C. R. Nelson, G. L. Gabor, Miklos, J. F. Abril, A. Agbayani, H.-J. An, C. Andrews-Pfannkoch, D. Baldwin, R. M. Ballew, A. Basu, J. Baxendale, L. Bayraktaroglu, E. M. Beasley, K. Y. Beeson, P. V. Benos, B. P. Berman, D. Bhandari, S. Bolshakov, D. Borkova, M. R. Botchan, J. Bouck, P. Brokstein, P. Brottier, K. C. Burtis, D. A. Busam, H. Butler, E. Cadieu, A. Center, I. Chandra, J. M. Cherry, S. Cawley, C. Dahlke, L. B. Davenport, P. Davies, B. d. Pablos, A. Delcher, Z. Deng, A. D. Mays, I. Dew, S. M. Dietz, K. Dodson, L. E. Doup, M. Downes, S. Dugan-Rocha, B. C. Dunkov, P. Dunn, K. J. Durbin, C. C. Evangelista, C. Ferraz, S. Ferriera, W. Fleischmann, C. Fosler, A. E. Gabrielian, N. S. Garg, W. M. Gelbart, K. Glasser, A. Glodek, F. Gong, J. H. Gorrell, Z. Gu, P. Guan, M. Harris, N. L. Harris, D. Harvey, T. J. Heiman, J. R. Hernandez, J. Houck, D. Hostin, K. A. Houston, T. J. Howland, M.-H. Wei, C. Ibegwam, M. Jalali, F. Kalush, G. H. Karpen, Z. Ke, J. A. Kennison, K. A. Ketchum, B. E. Kimmel, C. D. Kodira, C. Kraft, S. Kravitz, D. Kulp, Z. Lai, P. Lasko, Y. Lei, A. A. Levitsky, J. Li, Z. Li, Y. Liang, X. Lin, X. Liu, B. Mattei, T. C. McIntosh, M. P. McLeod, D. McPherson, G. Merkulov, N. V. Milshina, C. Mobarry, J. Morris, A. Moshrefi, S. M. Mount, M. Moy, B. Murphy, L. Murphy, D. M. Muzny, D. L. Nelson, D. R. Nelson, K. A. Nelson, K. Nixon, D. R. Nusskern, J. M. Pacleb, M. Palazzolo, G. S. Pittman, S. Pan, J. Pollard, V. Puri, M. G. Reese, K. Reinert, K. Remington, R. D. C. Saunders, F. Scheeler, H. Shen, B. C. Shue, I. Sidn-Kiamos, M. Simpson, M. P. Skupski, T. Smith, E. Spier, A. C. Spradling, M. Stapleton, R. Strong, E. Sun, R. Svirskas, C. Tector, R. Turner, E. Venter, A. H. Wang, X. Wang, Z.-Y. Wang, D. A. Wassarman, G. M. Weinstock, J. Weissenbach, S. M. Williams, T. Woodage, K. C. Worley, D. Wu, S. Yang, Q. A. Yao, J. Ye, R.-F. Yeh, J. S. Zaveri, M. Zhan, G. Zhang, Q. Zhao, L. Zheng, X. H. Zheng, F. N. Zhong, W. Zhong, X. Zhou, S. Zhu, X. Zhu, H. O. Smith, R. A. Gibbs, E. W. Myers, G. M. Rubin, and J. C. Venter, 2000 The Genome Sequence of Drosophila melanogaster. Science **287**: 2185–2195.

Chrostek, E., M. S. P. Marialva, S. S. Esteves, L. A. Weinert, J. Martinez, F. M. Jiggins, and L. Teixeira, 2013 Wolbachia variants induce differential protection to viruses in Drosophila melanogaster: a phenotypic and phylogenomic analysis. PLoS Genet. **9**: e1003896.

Clark, M. E., C. L. Anderson, J. Cande, and T. L. Karr, 2005 Widespread prevalence of Wolbachia in laboratory stocks and the implications for Drosophila research. Genetics **170**: 1667–1675.

Darby, A. C., A. Christina Gill, S. D. Armstrong, C. S. Hartley, D. Xia, J. M. Wastling, and B. L. Makepeace, 2014 Integrated transcriptomic and proteomic analysis of the global response of Wolbachia to doxycycline-induced stress. ISME J **8**: 925–937.

Dehal, P. S., M. P. Joachimiak, M. N. Price, J. T. Bates, J. K. Baumohl, D. Chivian, G. D. Friedland, K. H. Huang, K. Keller, P. S. Novichkov, I. L. Dubchak, E. J. Alm, and A. P. Arkin, 2010 MicrobesOnline: an integrated portal for comparative and functional genomics. Nucleic Acids Res. **38**: D396–400.

Hoffmann, A. A., M. Hercus, and H. Dagher, 1998 Population dynamics of the Wolbachia infection causing cytoplasmic incompatibility in Drosophila melanogaster. Genetics **148**: 221–231.

Huang, W., A. Massouras, Y. Inoue, J. Peiffer, M. Rmia, A. M. Tarone, L. Turlapati, T. Zichner, D. Zhu, R. F. Lyman, M. M. Magwire, K. Blankenburg, M. A. Carbone, K. Chang, L. L. Ellis, S. Fernandez, Y. Han, G. High-

nam, C. E. Hjelmen, J. R. Jack, M. Javaid, J. Jayaseelan, D. Kalra, S. Lee, L. Lewis, M. Munidasa, F. Ongeri, S. Patel, L. Perales, A. Perez, L. Pu, S. M. Rollmann, R. Ruth, N. Saada, C. Warner, A. Williams, Y.-Q. Wu, A. Yamamoto, Y. Zhang, Y. Zhu, R. R. H. Anholt, J. O. Korbel, D. Mittelman, D. M. Muzny, R. A. Gibbs, A. Barbadilla, J. S. Johnston, E. A. Stone, S. Richards, B. Deplancke, and T. F. C. Mackay, 2014 Natural variation in genome architecture among 205 Drosophila melanogaster Genetic Reference Panel lines. Genome Res. **24**: 1193–1208.

Mao, X., Q. Ma, C. Zhou, X. Chen, H. Zhang, J. Yang, F. Mao, W. Lai, and Y. Xu, 2014 DOOR 2.0: presenting operons and their functions through dynamic and integrated views. Nucleic Acids Res. **42**: D654–659.

Papafotiou, G., S. Oehler, C. Savakis, and K. Bourtzis, 2011 Regulation of Wolbachia ankyrin domain encoding genes in Drosophila gonads. Res. Microbiol. **162**: 764–772.

Pertea, M., K. Ayanbule, M. Smedinghoff, and S. L. Salzberg, 2009 OperonDB: a comprehensive database of predicted operons in microbial genomes. Nucleic Acids Res. **37**: D479–D482.

Richardson, M. F., L. A. Weinert, J. J. Welch, R. S. Linheiro, M. M. Magwire, F. M. Jiggins, and C. M. Bergman, 2012 Population genomics of the Wolbachia endosymbiont in Drosophila melanogaster. PLoS Genet. **8**: e1003129.

Si, Y., P. Liu, P. Li, and T. P. Brutnell, 2014 Model-based clustering for RNA-seq data. Bioinformatics **30**: 197–205.

Taboada, B., R. Ciria, C. E. Martinez-Guerrero, and E. Merino, 2012 ProOpDB: prokaryotic operon database. Nucleic Acids Res. **40**: D627–D631.
