# Supplementary material for: Dynamics of Wolbachia pipientis Gene Expression Across the Drosophila melanogaster Life Cycle
Source: G3 (Bethesda). 2015 Oct 23;5(12):2843–56. doi: 10.1534/g3.115.021931 (PMC4683655; doi:10.1534/g3.115.021931)
Supplement: Supporting Information [file supp_g3.115.021931_FigureS1.pdf]

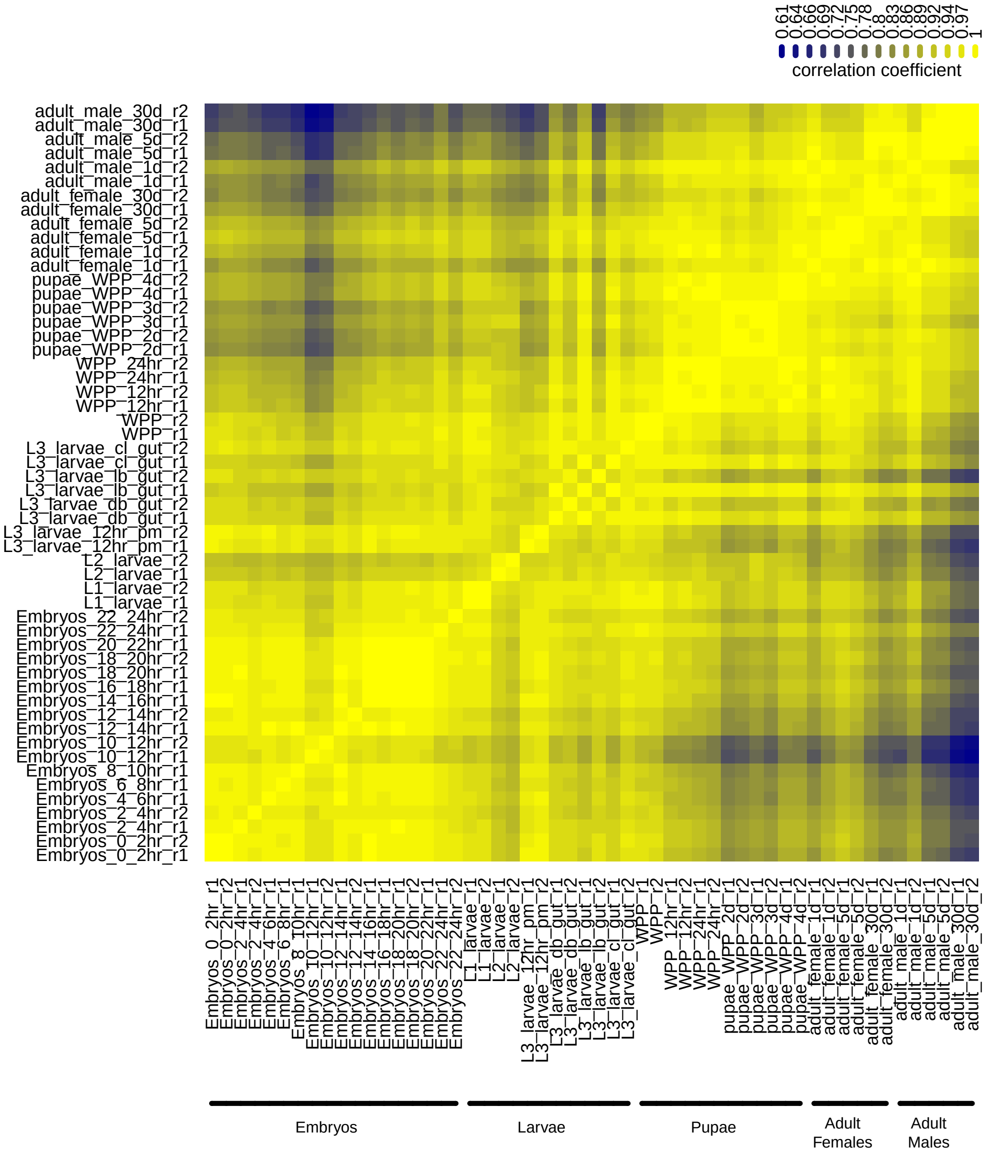
correlation coefficient
Embryos
Larvae
Pupae
Adult Females
Adult Males

Figure S1. ***Wolbachia*** **gene expression levels are highly-correlated across biological replicates and life cycle stages.**

Each cell in the heatmap represents a Pearson correlation coefficient of expression levels across all genes (in units of TPM) for a pair of samples in the ISO1 total RNA-seq dataset. Higher similarity among pairs of samples is represented by bright yellow and lower similarity by dark blue. All but six stages in the modENCODE total RNA-seq time course have biological replicates (Embryos 4-6 hrs, Embryos 6-8 hrs, Embryos 8-10 hrs, Embryos 14-16 hrs, Embryos 16-18 hrs, and Embryos 20-22 hrs). Replicate samples from the same stage were collected in two independent series, denoted by _r1 and _r2 suffixes. Correlation among biological replicates of the same stage is very high ($\geq$0.94), with the exception of late larval L3 stages (dark blue gut, light blue gut and clear gut) where samples from different stages of the same replicate series had higher correlation with each other than replicate samples from the same stage, leading to the observed checkerboard pattern. Two clusters spanning embryonic to white prepupal (WPP) stages, and late larval to adult stages, respectively, can be seen as square blocks of yellow spanning multiple stages.
