# Supplementary material for: Dynamics of Wolbachia pipientis Gene Expression Across the Drosophila melanogaster Life Cycle
Source: G3 (Bethesda). 2015 Oct 23;5(12):2843–56. doi: 10.1534/g3.115.021931 (PMC4683655; doi:10.1534/g3.115.021931)
Supplement: Supporting Information [file supp_g3.115.021931_FigureS2.pdf]

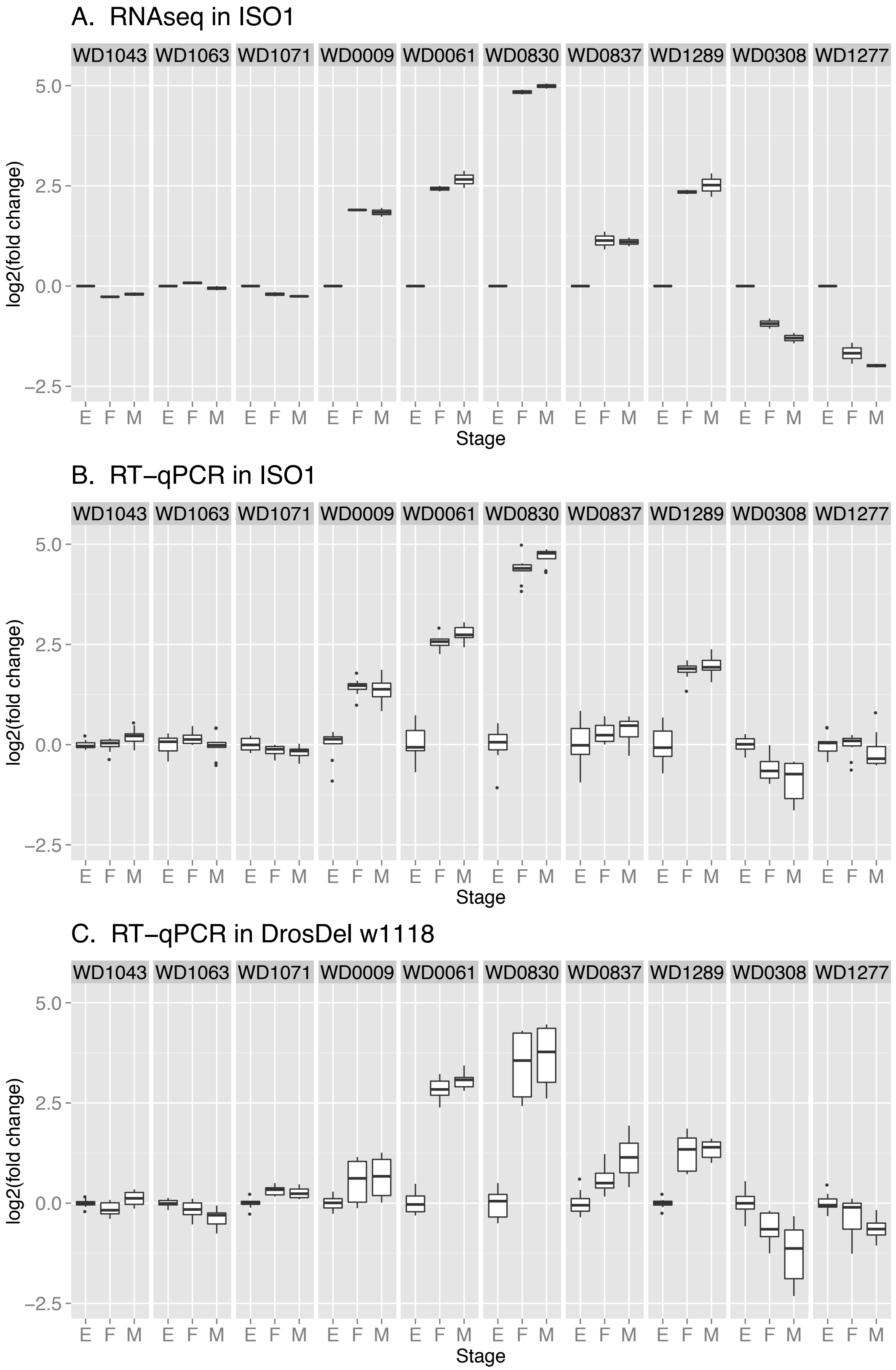

A. RNAseq in ISO1
WD1043
WD1063
WD1071
WD0009
WD0061
WD0830
WD0837
WD1289
WD0308
WD1277
5.0
2.5
0.0
−2.5
log2(fold change)
E F M
Stage
B. RT−qPCR in ISO1
C. RT−qPCR in DrosDel w1118

Figure S2. Confirmation of stably- and differentially-expressed genes by RT-qPCR.

(A) Relative expression based on RNA-seq in ISO1. (B) Relative expression based on RT-qPCR for ISO1. (C) Relative expression based on RT-qPCR for DrosDel w1118. Stages correspond to embryo 16-18 hrs [E], 1-day post-eclosion females [F], and 1-day post-eclosion males [M]. RNA-seq expression levels for each gene were based on TPMs and normalized relative to embryonic expression levels for that gene. RT-qPCR expression levels for each gene were normalized using the mean expression of three stably-expressed reference genes (WD1043, WD1063, WD1071) and are calculated relative to embryonic expression levels. Relative expression levels are shown as boxplots with black lines representing median values, boxes representing the interquartile range (IQR), whiskers representing the limits of values for samples that lie within 1.5 x IQR of the upper or lower quartiles, and dots representing samples that lie outside 1.5 x IQR of the upper or lower quartiles. In (A), there is one biological replicate for the embryonic stage and two replicates per stage for males and females. In (B) and (C), for each stage in each genotype, there are ten biological replicates from two independent collections (five replicates from each collection). Genes predicted to be stably-expressed, up-regulated (WspB/WD0009, WD0061, WD0830, WD0837, WD1289) or down-regulated (groES/WD0308, Hsp90/WD1277) between embryos and adults by RNA-seq showed expected patterns by RT-qPCR. Results of GLMs for differences in RT-qPCR expression levels between stages can be found in Table S3.
